# Double cytoplast embryonic cloning improves *in vitro* but not *in vivo* development from mitotic pluripotent cells in cattle

**DOI:** 10.3389/fgene.2022.933534

**Published:** 2022-09-28

**Authors:** Sarah Jane Appleby, Pavla Misica‐Turner, Fleur Catherine Oback, Arindam Dhali, Zachariah Louis McLean, Björn Oback

**Affiliations:** ^1^ Animal Biotech, AgResearch, Hamilton, New Zealand; ^2^ School of Science, University of Waikato, Hamilton, New Zealand; ^3^ School of Medical Sciences, University of Auckland, Auckland, New Zealand

**Keywords:** bovine, cloning, embryo (animal), pluripotent, cell cycle, mitotic

## Abstract

Cloning multiple animals from genomically selected donor embryos is inefficient but would accelerate genetic gain in dairy cattle breeding. To improve embryo cloning efficiency, we explored the idea that epigenetic reprogramming improves when donor cells are in mitosis. We derived primary cultures from bovine inner cell mass (ICM) cells of *in vitro* fertilized (IVF) embryos. Cells were grown feeder-free in a chemically defined medium with increased double kinase inhibition (2i^+^). Adding recombinant bovine interleukin 6 to 2i^+^ medium improved plating efficiency, outgrowth expansion, and expression of pluripotency-associated epiblast marker genes (*NANOG, FGF4, SOX2,* and *DPPA3*). For genotype multiplication by embryonic cell transfer (ECT) cloning, primary colonies were treated with nocodazole, and single mitotic donors were harvested by mechanical shake-off. Immunofluorescence against phosphorylated histone 3 (P-H3) showed 37% of nocodazole-treated cells in metaphase compared to 6% in DMSO controls (*P* < 1 × 10^−5^), with an average of 53% of P-H3-positive cells expressing the pluripotency marker SOX2. We optimized several parameters (fusion buffer, pronase treatment, and activation timing) for ECT with mitotic embryonic donors. Sequential double cytoplast ECT, whereby another cytoplast was fused to the first cloned reconstruct, doubled cloned blastocyst development and improved morphological embryo quality. However, *in situ* karyotyping revealed that over 90% of mitotic ECT-derived blastocysts were tetraploid or aneuploid with extra chromosomes, compared to less than 2% in the original ICM donor cells. Following the transfer of single vs. double cytoplast embryos, there was no difference between the two methods in pregnancy establishment at D35 (1/22 = 5% vs. 4/53 = 8% for single vs. double ECT, respectively). Overall, post-implantation development was drastically reduced from embryonic mitotic clones when compared to somatic interphase clones and IVF controls. We conclude that mitotic donors cause ploidy errors during *in vitro* development that cannot be rescued by enhanced epigenetic reprogramming through double cytoplast cloning.

## Introduction

Over the past two decades, somatic cell transfer (SCT) has virtually replaced embryonic cell transfer (ECT) in livestock cloning experiments. This has obscured the conceptual advantages of embryo-based approaches for accelerating genetic improvement by genome selection, modification, and multiplication (Wells et al., 2003a). First, embryo selection captures the most recent genetic gains, compared to the lag associated with somatic cloning from young or adult animals (Mclean et al., 2020); second, embryonic genomes are more amenable to complex modifications (Hockemeyer et al., 2009; Buecker et al., 2010; Hockemeyer et al., 2011); and third, embryonic donors are easier to reprogram, increasing cloning efficiency while reducing animal welfare issues and production costs (Heyman et al., 2002a; Misica-Turner et al., 2007). However, embryonic blastomeres are limited in numbers and technically more challenging to prepare than somatic donors (Wells et al., 2003a). With the advent of accurate marker- and sequence-based embryo selection, precise multi-editing, and serial embryo multiplication, there is a demand to improve ECT efficiency, especially by using embryonic pluripotent stem cells (or ePSCs (Verma et al., 2013)) as donors.

Under the right culture conditions, individual inner cell mass (ICM)–derived ePSCs remain capable of producing all adult cell types (Evans and Kaufman, 1981; Martin, 1981), even after homologous recombination (Capecchi, 2005) or gene editing (Mulas et al., 2019). Only cells engendering germline transmission after diploid (Bradley et al., 1984) or tetraploid embryo complementation (Nagy et al., 1990) are fully pluripotent and referred to as “naïve” PSCs (Nichols and Smith, 2009). Naïve PSCs can extensively self-renew and biologically amplify a selected genotype without acquiring genetic abnormalities (Suda et al., 1987). They can also be efficiently expanded from single cells, allowing *in vitro* multiplication of embryonic genotypes after genetic modification.

Attempts to isolate naïve ePSCs from cattle have so far failed (Oback et al., 2014). However, extensive chemical screening, together with a better understanding of pluripotency regulation in mice and humans, has produced so-called primed or “expanded potential” stem cells in pigs (Choi et al., 2019; Gao et al., 2019) and cattle (Bogliotti et al., 2018; Zhao et al., 2021). Under chemically defined culture conditions, these cells showed molecular and functional features of naïve pluripotency, including pluripotency gene expression, genome editing potential, and limited chimera contribution. Undifferentiated porcine and bovine ePSCs reached >40 passages while remaining karyotypically normal (Gao et al., 2019) and competent to form teratomas (Zhao et al., 2021). Similar results were reported for bovine ePSC-like cells (Bogliotti et al., 2018). These ePSCs engendered embryonic and extra-embryonic somatic cell lineages, but not the germline, in pig (Gao et al., 2019) and bovine (Zhao et al., 2021) chimeras.

Even though ePSCs have been used for ECT to generate blastocysts *in vitro*, no fully ePSC-derived farm animals have yet been generated (Sims and First, 1994; Campbell et al., 1996a; Saito et al., 2003; Xue et al., 2016; Zhao et al., 2021). In the mouse, the high apparent cloning efficiency of ES cells vs. somatic donors has been widely cited (Perry, 2004). However, this is confounded by the donor cell cycle stage, which affects both genetic and epigenetic aspects of nuclear reprogramming (Oback and Wells, 2007). Genetically, cell cycle coordination between the nuclear donor and enucleated recipient cell (cytoplast) ensures normal ploidy in the reconstructed embryo (Campbell et al., 1996b). G_0_/G_1_ and fully DNA-replicated G_2_/M-phase donors can produce blastocysts and offspring upon transfer into non-activated cytoplasts, whereas partially replicated S-phase donors cannot (Campbell et al., 1996b; Tani et al., 2001). Direct comparisons between genetically permissive cell cycle stages (G_0_/G_1_ and G_2_/M) in mouse ES cell donors have found no effect on reprogramming and cloning efficiency (Wakayama et al., 1999; Yabuuchi et al., 2004). During bovine SCT, G_0_ increased cloning efficiency compared to G_1_ cells (Kallingappa et al., 2016), but collectively, G_0_/G_1_ cells were reprogrammed similarly to G_2_/M donors (Tani et al., 2001). However, these findings contrast with multiple lines of evidence in frogs, suggesting that the nuclear reprogrammability of somatic donor cells is increased during mitosis (Egli et al., 2008). Following SCT into *Xenopus* oocytes, G_2_/M-phase donors showed dramatically increased transcriptional reprogramming of pluripotency genes when compared to interphase nuclei (Halley-Stott et al., 2014). This “mitotic advantage” is thought to be partially attributable to epigenetic effects, such as histone H2A deubiquitination, that accelerate the access of cytoplasmic reprogramming factors to mitotic chromatin. It was further suggested that the removal of most transcription factors from mitotic chromosomes increased their accessibility to reprogramming factors, allowing for rapid induction as soon as transcription resumes upon exit from mitosis (Halley-Stott et al., 2014). However, the impact on development was not investigated in these studies, and so the significance of the donor cell cycle on epigenetic reprogramming during cloning remains controversial.

Herein, we addressed the effect of donor cell mitosis on ECT-mediated reprogramming in cattle. We established and characterized chemically defined ePSC culture conditions in the presence of interleukin 6 (IL-6). Following optimized nocodazole arrest of primary ePSC cultures, we investigated reprogramming success after ECT with mitotically arrested donors. Following optimized sequential ECT, we found that mitotic embryonic donors produced cloned blastocysts at comparable rates to interphase donors. However, over 90% of mitotic ECT-derived blastocysts were karyotypically abnormal. Post-implantation development from embryonic mitotic clones was poor, indicating that underlying ploidy errors compromise *in vivo* survival.

## Materials and methods

Chemicals were supplied by Sigma-Aldrich (Auckland, New Zealand) and all embryo manipulations were carried out at 38.5°C unless indicated otherwise. Investigations were conducted in accordance with the regulations of the New Zealand Animal Welfare Act 1999.

### *In vitro* maturation of oocytes (IVM)

*In vitro* matured metaphase II (MII)-arrested oocytes were derived as described previously (Campbell et al., 1996b). Briefly, slaughterhouse ovaries were collected from mature cows, placed into saline (30°C), and transported into the laboratory within 2–4 h. Cumulus-oocyte complexes (COCs) were collected in Hepes-buffered medium 199 (H199) (Life Technologies; Cat.-No. 31100-035) containing 15 mM Hepes, 5 mM NaHCO_3_, and 0.086 mM kanamycin monosulfate, with 925 IU/ml heparin (CP Pharmaceuticals, United Kingdom) and 2% (w/v) fetal bovine serum (Life Technologies, United States) by aspirating 3–10 mm follicles into a 15 ml Falcon tube using an 18-gauge needle and negative pressure (40–50 mm Hg). Only COCs with a compact, non-atretic cumulus corona, and a homogenous ooplasm were selected for IVM. COCs were washed twice in H199 with 10% (v/v) FBS (H199-10) and once in bicarbonate-buffered medium M199 with 25 mM NaHCO_3_, 0.2 mM pyruvate, 0.086 mM kanamycin monosulfate, and 10% (v/v) FBS (B199-10). Ten COCs in 10 µl of B199-10 were transferred into a 40 µl drop of IVM medium B199-10 with 10 µg/ml ovine follicle-stimulating hormone (Ovagen; ICPbio), 1 µg/ml ovine luteinizing hormone (ICPbio), 1 µg/ml 17-ß-estradiol, and 0.1 mM cysteamine (2-mercaptoethylamine) in 6-cm dishes (Falcon 35-1007, Becton Dickinson Labware, Lincoln Park, NJ, United States) overlaid with paraffin oil (Sigma). Dishes were cultured in humidified 5% CO_2_ in air atmosphere. After IVM for 18–20 h, the cumulus-corona was dispersed by vortexing up to 180 oocytes in 500 µl of 1 mg/ml bovine testicular hyaluronidase in Hepes-buffered synthetic oviduct fluid (HSOF) (107.7 mM NaCl, 7.15 mM KCl, 0.3 mM KH_2_PO_4_, 5 mM NaHCO_3_, 3.32 mM sodium lactate, 0.069 mM kanamycin monosulfate, 20 mM Hepes, 0.33 mM pyruvate, 1.71 mM CaCl_2_.2H_2_O, and 3 mg/ml fatty-acid free bovine albumin (ABIVP; ICPbio) followed by three washes in HSOF containing 0.1 mg/ml cold soluble PVA (M_r_: 10–30,000) (H199-PVA). For zona-free CT, oocytes with a first polar body were washed three times in H199-PVA before removal of the zona pellucidae by pronase (5 mg/ml in H199)*.*

### *In vitro* production (IVP) of embryos

*In vitro* matured oocytes were derived from slaughterhouse ovaries of mixed-breed dairy cows and fertilized with frozen–thawed spermatozoa for 22–24 h (Schurmann et al., 2006). Briefly, spermatozoa were prepared from frozen–thawed semen of a commercial sire with proven fertility *in vitro*. Two 0.25 ml straws were layered on a Percoll gradient (45%:90%), and motile spermatozoa were collected after centrifugation at 700 *g* for 20 min at room temperature. Sperm concentration was adjusted to 1 × 10^6^ sperm ml^−1^ and oocytes inseminated 22–24 h post start of maturation in 50 μl IVF SOF. Presumptive zygotes were washed in HSOF after 18–24 h and cultured for 7 days (D0: fertilization) in sequential early and late AgR-SOF (ESOF/LSOF) medium (Wells et al., 2003b). Embryo cultures were overlaid with mineral oil and kept in a humidified modular incubation chamber (ICN Biomedicals Inc., Aurora, OH, United States) gassed with 5% CO_2_, 7% O_2_, and 88% N_2_. All embryos were assigned a developmental stage (early, mid, expanded, or hatched blastocyst) and morphological grade (Robertson et al., 2011) by the same person throughout this study (BO).

### Isolation and culture of ICMs

Primary ICM cells were isolated from IVP embryos cultured for 8 days (D8) by immunosurgery (Verma et al., 2013). Following zona pellucida removal with 0.5% pronase, embryos were washed in Hepes-buffered transfer LSOF with 0.1 mg/ml cold soluble PVA (M_r_: 10–30,000) (“THSOF-PVA”). Groups of 8–10 embryos were incubated in 1:4 rabbit anti-bovine serum (Cat-No. B3759) for 40 min at 38.5°C, washed twice with THSOF-PVA, and placed into 1:4 guinea pig complement (Cat-No. S1639) for 15–30 min. Isolated ICMs were individually seeded on eight-well chamber slides coated overnight with gelatin (1 mg/ml in PBS) followed by natural mouse laminin (Life Technologies, Auckland, New Zealand) for 1 h at 5–7 μg/cm^2^. Cells were cultured in 300 μl N_2_B_27_ “2i^+^” based media, comprising DMEM/F12 (Thermo-Fisher, New Zealand) + N2 (Thermo-Fisher, New Zealand), mixed 1:1 with Neurobasal medium (Thermo-Fisher, New Zealand) + B27 (Thermo-Fisher, New Zealand) and 1 mM L-glutamine [“N2B27”] and supplemented with 10 µM forskolin (Cayman Chemical, United States), MAP2K inhibitor PD0325901 (10 mM stock, 0.4 μM final concentration, Stemgent, Cambridge, MA, United States) and GSK3B inhibitor CHIR99021 (10 mM stock, 3 μM final concentration, Stemgent) (“2i+”) (McLean et al., 2014) for 6 days before characterization or use in ECT. For some experiments, the medium was supplemented with 10 ng/ml human recombinant leukemia inhibitory factor (LIF; ORF Genetics, Kópavogur, Iceland), 10 ng/ml oncostatin M (OSM; Prospec, New Zealand), or 10 ng/ml bovine recombinant IL-6 (Kingfisher Biotech Inc., MN, United States). The colony area was determined using polygon selection and measure functions in ImageJ 1.45 s.

### nCounter analysis

For each treatment, pools of primary ICM cultures from three independent experiments were collected (*N* = 27 and *N* = 24 colonies for DMSO vs. 2i+/IL6, respectively), washed in PBS/PVA, and snap–frozen in liquid nitrogen. After adding 5 µl of *RNA*GEM^™^ Tissue *PLUS* (ZyGEM, New Zealand) solution, RNA was extracted at 75°C for 5 min. Gene expression of 169 targets associated with interleukin signaling, early embryonic development, and/or pluripotency (Supplementary Table S1) was quantified with an nCounter Analysis System (NanoString Technologies, Seattle, WA, United States). Sequence-specific target enrichment and hybridization of capture and reporter probes to the pre-amplified sample were carried out according to NanoString’s standard protocols (Kulkarni and Ausubel, 2011). Individual fluorescent barcodes were counted by the nCounter Digital Analyzer. To determine true counts and minimize background, the highest of eight unique internal negative control counts for each sample and each gene was subtracted from the raw counts before normalization (Supplementary Table S2). A normalization factor was calculated based on the product of 1) the geometric mean of five housekeeping genes (*GAPDH*, *PPIA*, *ACTB*, *YWHAZ,* and *PGK1*) and 2) the averages of the six exogenous spikes of known mRNA concentration. Each sample’s unique normalization factor was then applied to all target gene counts (Supplementary Table S2). Normalized counts for each treatment were averaged and log ratios over DMSO were calculated in Excel for generating volcano plots and presenting fold changes.

### RNA extraction and RT-qPCR

Grade 1–2 D8 blastocysts were lysed in 10 µl *RNA*GEM^™^ Tissue *PLUS* (with 0.5 µl *RNA*GEM^™^) and cDNA synthesized as described (Misica-Turner et al., 2007). Reverse transcriptase was omitted in one sample each time a batch was processed for cDNA synthesis (“-RT”). Primers were designed using NCBI/Primer-BLAST (Supplementary Table S3) and synthesized by Integrated DNA Technologies (IDT, IA, United States). For RT-qPCR, a LightCycler^©^ 2.0 (Roche, New Zealand) was used. All nCounter^©^ validation experiments were performed with the LightCycler^©^ FastStart DNA MasterPLUS SYBR Green I Kit. The ready-to-use “Hot Start” reaction mix consisted of 0.4 µl of each primer (10 µM), 2.0 µl master mix, 5.2 µl DEPC water, and 1.0–2.0 µl cDNA template. The following four-segment program was used: *1*) denaturation (10 min at 95°C); *2*) amplification and quantification (20 s at 95°C, 20 s at 55–60°C, followed by 20 s at 72°C with a single fluorescent measurement repeated 45 times); *3*) melting curve (95°C, then cooling to 65°C for 20 s, 0.2°C s^−1^ to 95°C while continuously measuring fluorescence); and *4*) cooling to 4°C. For relative quantification, external standard curves were generated from serial 5-log dilutions for each gene in duplicate. One high-efficiency curve (3.6 ≥ slope ≥ 3.1, *R* ^2^ > 0.99) was saved for each target gene and imported for relative quantification as described (Misica-Turner et al., 2007). Product identity was confirmed by gel electrophoresis and melting curve analysis. Assays were optimized to ensure a single melting peak corresponded to the correct PCR product size and the absence of primer-dimer formation.

### Immunofluorescence (IF)

The following antigens were analyzed: SOX2 (AF 2018, R&D Systems, Minneapolis, MN, United States), NANOG (eBioscience 14-5768, San Diego, CA, United States), phosphorylated (p) histone (H) 3 (Upstate #06–570), and Ki-67 (Abcam #15580). Cells were fixed in 4% (w/v) PFA/4% (w/v) sucrose in PBS for 15 min at 4°C, washed three times in PBS, quenched in 50 mM NH_4_Cl in PBS for 10 min, permeabilized in 0.1% (v/v) Triton X-100 in PBS for 10 min at room temperature, and blocked in 5% donkey or goat serum or 5% BSA in PBS for at least 30 min. Primary antibodies were incubated overnight at 4°C, washed in PBS, and incubated with Alexa Fluor^®^ 488 or 568 donkey anti-mouse, -rat, -rabbit, or -goat secondary IgG antibodies (all Life Technologies) for 30 min at 37°C. All antibodies were diluted in blocking buffer. DNA replication was assessed using a click-iT^®^ 5-ethynyl-2′-deoxyuridine (EdU) assay (Thermo-Fisher, New Zealand), and cells stained without EdU labeling served as a negative control. DNA was counterstained with 5 µg/ml Hoechst 33342. Preparations were washed thrice in PBS and once in H_2_O before mounting (ProLong Diamond Antifade, Life Technologies, United States). Images were taken on an epifluorescence microscope (AX-70, Olympus, Auckland, New Zealand) equipped with a Spot RT-KE slider CCD camera (Diagnostics Instruments Inc., Sterling Heights, MI, United States).

### Preparing nuclear donor cells

For SCT, two bovine fibroblast lines were used: bovine embryonic fibroblasts (BEF14) from a D46 embryo and LJ801 adult ear skin fibroblasts (Kallingappa et al., 2016). For quiescent donors, cells were obtained by culture in a medium containing 0.5% fetal bovine serum (FBS) for 5 days and harvested by trypsinization (Kallingappa et al., 2016). A mitotic shake-off method was used to isolate mitotic cells for cloning (Kallingappa et al., 2016). Cells were seeded at 2.5 × 10^4^ per cm^2^ for 1–2 days prior to SCT. On the day of SCT, cells were washed once with pre-warmed PBS and cultured for 1 h in DMEM/F12–0.5% FBS with 0.1 µM nocodazole. Mitotic cells were dislodged by gently tapping, aspirating the medium, and centrifugation for 3 min at 161 *g* at room temperature. The supernatant was removed, and cells were resuspended in H199 + 0.5% FCS with 0.1 µM nocodazole. ePSCs were cultured for 5–7 days before ECT. Prior to ECT, colonies were cultured in a 2i+/IL-6 medium containing 0.1–1.67 µM nocodazole for 4–22 h. On the day of ECT, single cells were isolated using chemical and enzymatic dissociation. Media was aspirated from the chamber and replaced with 200 µl of pre-warmed 0.5 mg/ml dispase. After 1–2 min, colonies dislodged from the chamber and were incubated in pronase for 5 min before being transferred to dissociation media (HSOF-Ca-BSA + 1 mg/ml PVA + 20% EDTA + 5 µg/ml cytochalasin B with nocodazole). Cells were dissociated into single-cell suspension by triturating colonies through a mouth pipette with increasingly smaller diameters, and the largest cells within the population were selected for ECT.

### Cloning and artificial activation

Cloning was carried out by adapting our bovine zona-free SCT standard operating procedure (Oback and Wells, 2003) for ECT and “embryonic cell double cytoplast” (ECDC) transfer. Donors and donor-cytoplast couplets were kept in the same nocodazole concentration used for cell synchronization until direct current (DC) electrical fusion (2 kV/cm, 2 × 10 µs square DC pulses delivered 1 s apart). Double cytoplast reconstructs were produced by fusing another MII cytoplast to the first reconstruct within 1 h (1.5 kV/cm, 2 × 10 µs pulses) in hypo-osmolar fusion buffer (165 mM mannitol, 50 µM CaCl_2_, 100 μM MgCl_2_, 500 µM Hepes, 0.05% bovine albumin [ABIVP, ICP], pH 7.3). Fused reconstructed embryos were washed once in HSOF-Ca+10% FBS and transferred into ESOF-Ca+10% FBS droplets. Reconstructed embryos were artificially activated within approximately 1 h post-fusion, using a combination of 5 µM ionomycin and 2 mM 6-dimethylaminopurine (6-DMAP). Reconstructs were kept singularly in drops of 5 μl. After 4 h in 6-DMAP, the reconstructs were washed twice in HSOF and transferred into ESOF culture medium droplets. Embryos were cultured singularly in 5 μl drops for 7 days, following the biphasic ESOF/LSOF media.

### Karyotyping

Three methods were used for spreading chromosomes. *1*) Somatic cells were nocodazole-arrested for 1 h and dropped onto slides to spread chromosomes (“standard karyotyping”). *2*) ePSCs were nocodazole-arrested overnight and karyotyped *in situ* by evaporating fixative from the eight-well chamber to spread chromosomes. A BEF control was performed to validate this procedure (“*in situ* karyotyping”). *3*) Embryos were synchronized with 1.67 µM nocodazole for 3–4 h and spread by *in situ* karyotyping. For standard cell karyotyping, pelleted cells were resuspended in a hypotonic 0.56% KCl solution at 37°C for 15 min to induce nuclear swelling and fixed in −20°C methanol:acetic acid (3:1) at 4°C for 30 min. Washing with fresh fixative was repeated twice before resuspending the pellet in 500 µl of ice-cold fixative and spreading onto chilled microscope slides. For *in situ* karyotyping, colonies were incubated in 0.9% sodium citrate for 20 min at 4°C followed by ice-cold fresh 3:2:1 methanol:acetic acid:water fixative for 2–3 min. Fixative was removed from the chamber and allowed to dry for at least 2 h on a warm stage before staining. Embryos were incubated in 0.9% sodium citrate + 0.1 mg/ml PBS/PVA for 20 min at 4°C. Using a finely pulled Pasteur pipette coated with dimethylpolysiloxane, embryos were fixed and dried as per *in situ* cell karyotyping. All slides were stained with 5% KaryoMAX^®^ Giemsa in Gurr buffer, pH 6.8 for 15 min, followed by washing under a gentle stream of tap water. Metaphase spreads were photographed using a ×100 oil immersion objective and a Spot RT-KE slider camera.

### Embryo transfer and pregnancy monitoring

Total embryo development to compacted morula and blastocyst stages was assessed on D7, and grade 1 to 3 (B^1-3^) blastocysts (Robertson et al., 1998) were selected for embryo transfer (ET). Recipient cows were synchronized as described (Oback and Wells, 2003). On D7 following estrus (estrus = D0 = day of ECT), a single blastocyst in Embryo Hold media (AgR) was loaded per 0.25 ml straw (Cryo-Vet, France) and transferred non-surgically into the uterine lumen ipsilateral to the corpus luteum. Using ultrasonography (Aloka SSD-500 scanner with a 5 MHz linear rectal probe, Austria), the pregnancy status of recipient cows was determined on D35 of gestation. Development throughout gestation was monitored approximately every 30 days, from D35 to D90, by ultrasonography.

### Statistical analysis

All values are presented as mean ± SEM unless indicated otherwise. Statistical significance was determined using the two-tailed *t*-test with equal variance or Fisher exact test for independence in 2 × 2 tables. We provide exact *p*-values where possible and indicate arbitrary thresholds for *p*-values where practical (Wasserstein et al., 2019; Wasserstein and Lazar, 2016). Logs of nCounter and qPCR gene expression data were analyzed using the residual maximum likelihood method in GenStat^®^ (16th Edition), with treatments as fixed effects and runs and samples within the run as random effects. Values are presented as mean ± SEM of the log ratios of gene expression relative to the DMSO control.

## Results

### 2i+/IL-6 medium shifts gene expression from hypoblast to epiblast signature

We first validated that commercially available recombinant cytokines of the IL-6 family stimulate phosphorylation (P) of STAT3 tyrosine (Y) 705 (Huang et al., 2014) in bovine cells. Using an antibody against pSTAT3^Y705^, we found that LIF, OSM, and IL-6 were functionally active and induced pSTAT3 in serum-starved embryonic fibroblasts (Supplementary Figure S1A). ICM plating efficiency after 1 week in culture was improved by LIF, OSM, or IL-6 with little difference between the three cytokines (Supplementary Figure S1B). Likewise, the size of the primary colony was improved by LIF, OSM, or IL-6 but showed no difference between the three cytokines (Supplementary Figure S1C). Omitting PD03 from the medium increased primary colony area, while combining IL-6 with OSM and LIF did not have an additive effect on colony growth (Supplementary Figure S1D). LIF is not robustly expressed in blastocysts (Eckert and Ni emann, 1998), while IL-6 and its receptors are abundantly detectable in bovine blastocysts by PCR (Wooldridge and Ealy, 2019) and digital expression profiling (McLean et al., 2014). Since there were no differences between the three cytokines in generating primary outgrowth cultures, we used IL-6 for subsequent studies. On average, 93 ± 5% of ICMs were immunosurgically isolated from the blastocyst and 94 ± 2% of ICMs attached to the substrate, resulting in 87 ± 6% of viable primary colonies after 1 week (*N* = 105, *n* = 5, Supplementary Figure S1E).

**FIGURE 1:**
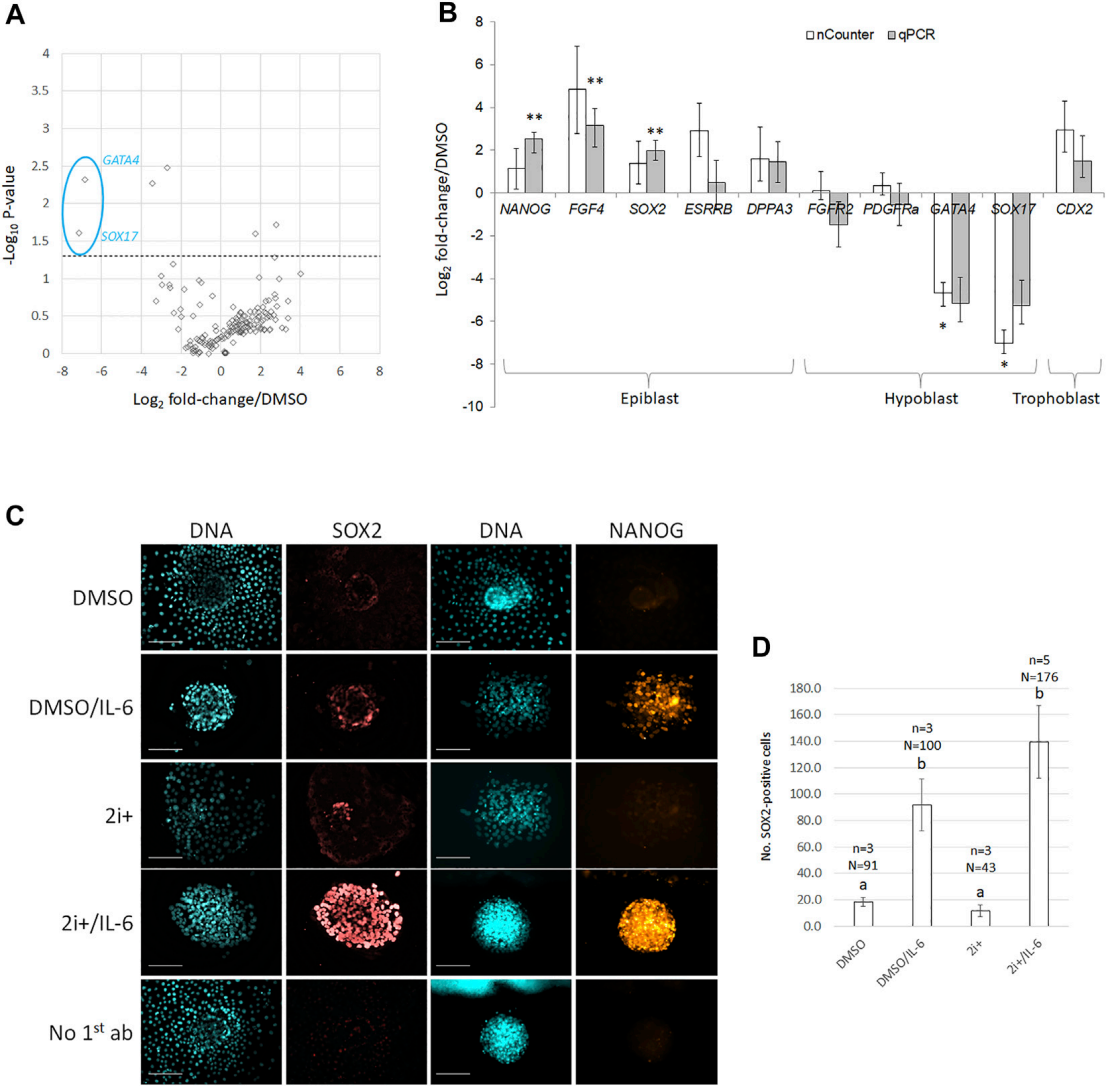
2i+/IL-6 reduces hypoblast and increases epiblast marker expression on mRNA and protein levels. **(A)** Volcano plots of gene expression changes. Biological fold changes were normalized on the DMSO control. A stippled line indicates *p* = 0.05 from a two-tailed *t*-test. **(B)** Comparison of nCounter vs. qRT-PCR analysis for putative lineage-specific marker genes. cDNA was extracted from pools of grade 1–2 D8 blastocysts cultured in DMSO- vs. inhibitor-containing medium. Target gene values are expressed as fold change over DMSO control; error bars indicate the mean ± SE of the log ratios of gene expression relative to the DMSO control. * and ** bars differ *p* < 0.05 and *p* < 0.01, respectively, from DMSO control. **(C)** Immunofluorescence against pluripotency-related epiblast markers (SOX2 and NANOG) and DNA (Hoechst 33342) on primary ICM outgrowth cultures in 2i+ and 2i+/IL6 cultures vs. DMSO controls. Scale bar = 100 μm. **(D)** Quantification of SOX2-positive nuclei per colony under the same culture conditions as in **(C)**; error bars indicate mean ± SE; a and b different superscripts indicate *p* < 0.05 from two-tailed *t*-test.

To correlate changes in colony size with changes in lineage segregation, we analyzed gene expression changes by multiplexed transcriptional profiling with the nCounter Analysis System (Geiss et al., 2008). We simultaneously quantified 169 different transcripts in 2i+/IL-6-treated primary cultures, including comprehensive profiling of interleukin signaling-related components (receptors/ligands), pluripotency-related, and lineage-specific candidate transcripts, as well as 10 housekeeping genes. Hypoblast-specific factors *GATA4* and *SOX17* were downregulated in 2i+/IL-6 (Figure 1A, *p* < 0.05), but none of the trophoblast markers (*CDX2*, *EOMES*, *GATA3*, *ID2*, *IFNT*, *KRT8*, *PDGFA*, and *TEAD4*) were significantly changed. From 76 interleukin-related genes, 21 (=28%) showed low (0–10 counts), 17 (=22%) medium (10–100 counts), 16 (=21%) high (100–1,000 counts), and 22 (=29%) very high (>1,000 counts) in the 2i+/IL6 group. Another 16 (=21%) genes were highly abundant in 2i+/IL-6 (*CD9*, *CXCR1*, *IL1RL1*, *IL2Ra*, *IL6R*, *IL11Ra*, *IL17RB*, *IL17F*, *IL18*, *IL20Rb*, *IL22Ra*, *IL23Ra*, *IL25*, *IL31*, *IL32*, and *IL37*), but only CD9 was highly abundant in both groups.

To validate the nCounter results, we followed up several candidates by RT-qPCR (Figure 1B). This confirmed robust upregulation of *NANOG*, *FGF4*, and *SOX2* (6-, 9-, and 4-fold, respectively, *p* < 0.001) with concomitant downregulation of *GATA4* and *SOX17* (36- and 39-fold, respectively, *p* < 0.001 and *p* < 0.05) in 2i+/IL-6. Trophoblast marker *CDX2* was not significantly changed.

To validate the effect on pluripotency-related epiblast markers, we evaluated the abundance of SOX2 and NANOG proteins by immunofluorescence (Figure 1C). Both markers were qualitatively upregulated by IL-6, in particular, in the presence of 2i+. Upregulation of SOX2 expression depended on IL-6 addition and was not induced by the 2i+ medium alone (Figure 1D). We conclude that 2i+/IL-6 stimulated robust expression of pluripotency-related markers.

### Arresting ICM-derived donor cells in metaphase

Recipient and donor cell cycles must be synchronized to maintain normal ploidy and allow epigenetic reprogramming. For cloned embryos to develop from mitotically arrested donor cells, we first optimized nocodazole synchronization time and concentration. Primary ICM-derived colonies were treated for 4 h or 21 h with 1.67 µM nocodazole to generate sufficient mitotic donor cells for ECT. Cells in mitosis were assessed by Hoechst stain to identify condensed chromatin (Supplementary Figure S2A). No difference was observed between DMSO vehicle controls and colonies arrested with nocodazole for 4 h (8 ± 9% and 12 ± 5%, respectively), but incubation with nocodazole for 21 h arrested nearly half the colony in mitosis (49 ± 1%; *p* < 0.05, Supplementary Figure S2B).

To optimize nocodazole concentration, we tested a range of 0.1–1.67 µM. Colonies were arrested for 24 h prior to being stained with Hoechst (Supplementary Figure S2C). Compared with 0.1 µM (158/572 = 28 ± 3%; *N* = 4 colonies), there was a higher number of nuclei with condensed DNA at 0.5 µM nocodazole (169/413 = 41 ± 6%, *p* < 0.05; *N* = 2) and 1 µM nocodazole (225/495 = 45 ± 2%, *p* < 0.01; *N* = 3, Supplementary Figure S2D). Incubation with the commonly reported 1.67 µM nocodazole produced a higher proportion of cells arrested (91/244 = 37 ± 5%; *N* = 3), but this was not significant compared with 0.1 µM (*p* = 0.08). All nocodazole concentrations produced metaphase-arrested cells compared to a DMSO-only control (13/171 = 8 ± 9%, *p* < 0.05; *N* = 2). As no significant differences were observed between 0.5, 1, and 1.67 µM, we used 0.5 µM nocodazole for 18–22 h in subsequent cloning experiments.

**FIGURE 2:**
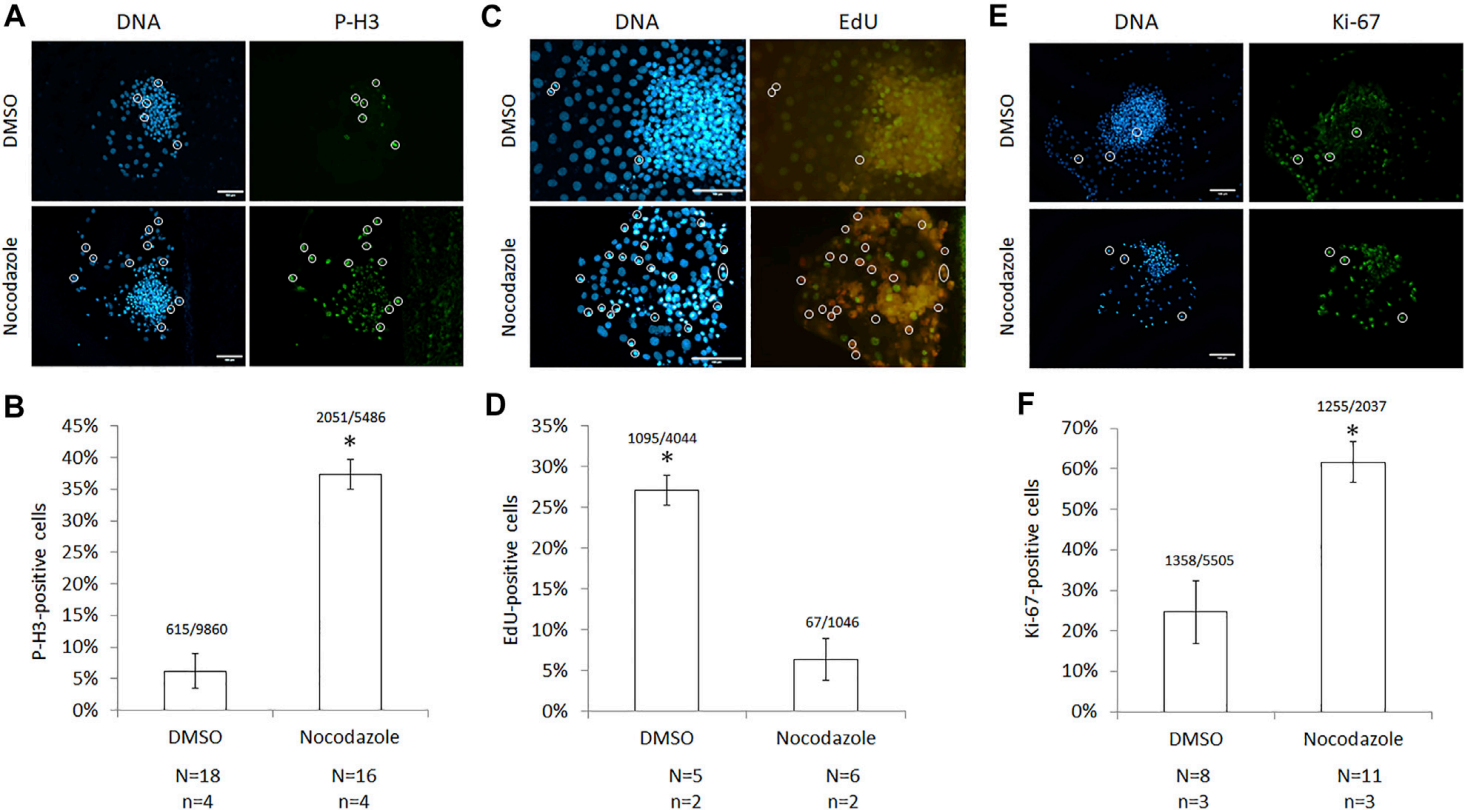
Cell-cycle characterization of nocodazole-arrested ICM cells. **(A)** Images and **(B)** quantification of cells with phosphorylated histone 3 (P-H3) following nocodazole synchronization for 18–22 h. A total of 15,346 Hoechst-stained nuclei (DNA) were counted. **(C)** Images and **(D)** quantification of cells with 5-ethynyl-2′-deoxyuridine (EdU) incorporation after a 30 min label following synchronization; 5,090 Hoechst-stained nuclei were counted. **(E)** Images and **(F)** quantification of cells expressing Ki-67 following synchronization; 7,542 Hoechst-stained nuclei were counted. For all panels, circles identify mitotic nuclei, scale bar = 100 μm, error bars = SEM, *N* = colonies counted per treatment*, n =* technical replicates, and * = *p* < 0.00001 from two-tailed *t*-test compared with DMSO.

For quantifying mitotic arrest *in situ* more accurately, cells were stained with antibodies against phosphorylated histone 3 (Ser10), which specifically detects mitotic chromosomes (Figure 2A). Nocodazole-arrested colonies had a higher proportion of P-H3-positive, mitotic cells than DMSO controls (37 ± 2% and 6 ± 3%, respectively; *p* < 0.01; Figure 2B). Per treatment, 16–18 colonies (*n* = 4 replicates) were counted with an average of 444 cells per colony (24–1,115 cells per colony, total cells *N* = 15,346). Since S-phase cells are incompatible with cloned embryo development, DNA-replicating cells were quantified after 30 min EdU incorporation (Figure 2C). Nocodazole-arrested colonies had a lower proportion of EdU-positive cells than DMSO controls (6 ± 2% and 27 ± 2%, respectively; *p* < 0.01; Figure 2D). Per treatment, four to five colonies (*n* = 2 replicates) were counted with an average of 530 cells per colony (73–1,196 cells per colony, total cells counted *N* = 4,766). Last, the proliferative fraction of cells in each colony was evaluated by staining for Ki-67 protein (Figure 2E). Nocodazole-arrested colonies had a higher number of Ki-67 positive cells than DMSO controls (62 ± 5% and 25 ± 8%, respectively; *p* < 0.01; Figure 2F). Per treatment, 8–11 colonies (*n* = 3) were counted with an average of 185 cells per nocodazole colony (71–354 cells per colony, *N* = 11 colonies, total cells counted *N* = 2,037) and 688 cells per DMSO colony (283–1,191 cells per colony, *N* = 8 colonies, total cells counted *N* = 5,505). Correct identification of cells in mitosis was established by DNA-staining isolated ePSCs and couplets: 66% (*N* = 280) of isolated single ePSCs were mitotic, while 94% (*N* = 18) of selected donor cells attached to a cytoplast were mitotic (data not shown). We conclude that nocodazole enabled the isolation of single, mitotically arrested embryonic donor cells.

### Expression of pluripotency markers in mitotic embryonic donor cells

**FIGURE 3:**
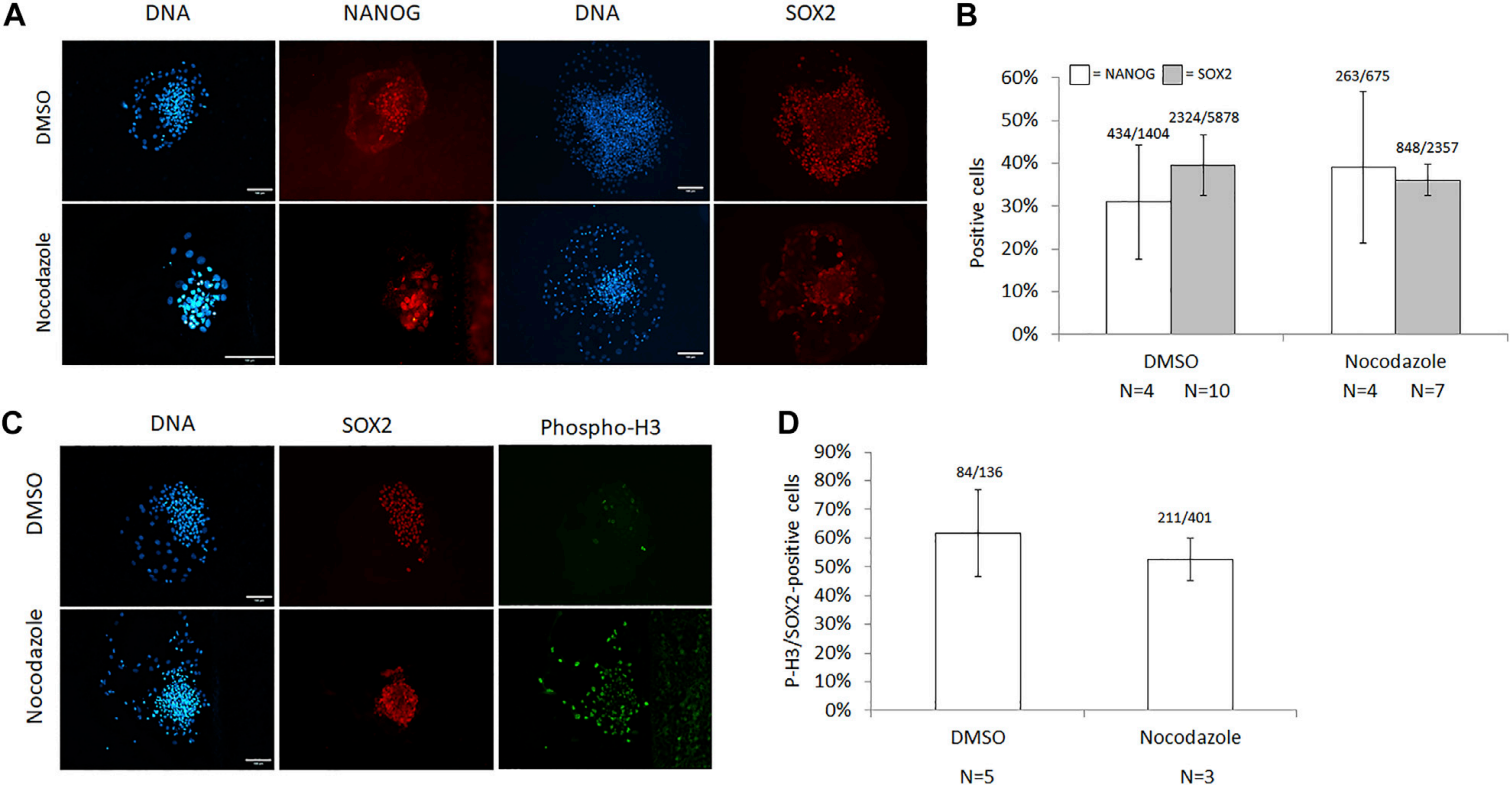
Characterization of pluripotency markers in nocodazole-synchronized ICM cells. **(A)** Images and **(B)** quantification of cells expressing NANOG and SOX2 following synchronization with nocodazole for 18–22 h. The number of Hoechst-stained nuclei (DNA) for NANOG (white bars) and SOX2 (grey bars) quantification was 2,079 (*p* = 0.63) and 8,235 (*p* = 0.26), respectively. **(C)** Images and **(D)** quantification of cells co-expressing P-H3 and SOX2 following synchronization with nocodazole for 18–22 h, and 537 P-H3-stained nuclei were counted (*p* = 0.45). Scale bar = 100 μm, error bars = SEM, *N* = colonies counted per treatment, and *p*-values from two-tailed *t*-test compared with DMSO.

The presence of pluripotency markers NANOG and SOX2 was assessed in nocodazole-arrested cells and DMSO controls (Figure 3A). There was no significant difference in the proportion of NANOG- or SOX2-positive cells in nocodazole-treated vs. DMSO colonies (39 ± 18% vs. 31 ± 13%, *p* = 0.63; and 36 ± 4% vs. 40 ± 7%, *p* = 0.27, respectively, Figure 3B). For NANOG, four colonies (*n* = 2 biological replicates) were counted with an average of 169 cells per nocodazole colony (58–356 cells per colony, total cells counted *N* = 675) and 351 cells per DMSO colony (34–639 cells per colony, total cells counted *N* = 1,404). For SOX2, 7–10 colonies (*n* = 2) were counted with an average of 218 cells per nocodazole colony (82–371 cells per colony, total colonies counted *N* = 7, total cells counted *N* = 2,357) and 330 cells per DMSO colony (54–669 cells per colony, total colonies counted *N* = 10, total cells *N* = 5,878). Last, we examined the mitotic fraction of the cell population’s SOX2 expression (Figure 3C). Over half of the cells positive for P-H3 were also positive for SOX2 in both DMSO and nocodazole-arrested colonies (62 ± 15% and 53 ± 7%, respectively; *p* = 0.45; Figure 3D). Per treatment, three to five colonies were counted with an average of 134 P-H3-positive cells per nocodazole colony (121–157 cells per colony, total colonies counted *N* = 3, total cells counted *N* = 401) and 34 cells per DMSO colony (16–58 cells per colony, total colonies counted *N* = 5, total cells counted *N* = 136).

### ICM-derived mitotic donors support cloned blastocyst development

Nocodazole-arrested mitotic donors were used as donors for zona-free ECT into MII cytoplasts. We optimized several parameters regarding the ECT method itself and artificial activation timing. First, we compared the osmolarity of the fusion buffer for lysis of both somatic (BEF14) and ICM-derived mitotic donor cells (Supplementary Figure S3A). In hypo-osmolar (164 mOsm) fusion buffer, 22 ± 2.5% of mitotic donor cells lysed prior to administering the fusion pulse (*n* = 3 experiments; *N* = 303 couplets). Iso-osmolar (270 mOsm) fusion buffer decreased the lysis rate to 9 ± 0.4% (*n* = 2; *N* = 191, *p* < 0.01). Second, treating interphase somatic cells with pronase for 5 min prior to attaching the cytoplast has improved fusion efficiency (Oback and Wells, 2003) and was compared for mitotic donors (Supplementary Figure S3B). In hypo-osmolar fusion buffer, there was no significant difference in fusion rate between untreated and pronase-treated BEF14 cells after mitotic shake-off (65 ± 7% and 75 ± 10%, respectively; *p* = 0.15; *n* = 3, *N* = 260). By contrast, in iso-osmolar fusion buffer, the fusion rate of pronase-treated cells was higher than that of untreated cells (72 ± 6% and 44 ± 5%, respectively; *p* < 0.01; *n* = 7, *N* = 351). Since pronase treatment combined with iso-osmolar, rather than hypo-osmolar, fusion buffer resulted in more SCT reconstructs (72 × 91 = 66% vs. 75 × 78 = 59%), this condition was used for subsequent cloning experiments. Overall, it resulted in acceptable first fusion rates during ECT (608/786 = 77.4%, *n* = 13 cloning runs). Third, the chemical activation procedure and timing were altered to achieve correct ploidy in the ECT reconstruct. Since DMAP prevents polar body extrusion and ploidy correction, we delayed the interval between ionomycin activation and DMAP incubation to facilitate the expulsion of a pseudo-polar body following mitotic ECT. In pilot experiments, parthenogenotes were activated with ionomycin and scored for extrusion of a polar body (Supplementary Figure S3C). Extrusion was assessed every 15 min and plateaued after around 90 min (*n* = 8, *N* = 557 embryos) at over 80% (Supplementary Figure S3D). Embryos that had extruded a polar body after 90 or 120 min were cultured in DMAP for 4 h, and development was assessed on D7 (Supplementary Figure S3E). Their development was compared with embryos activated under standard conditions, which was ionomycin followed by 4 h culture in DMAP within less than 10 min. Total and high-grade blastocyst development from standard controls (56 ± 10% and 36 ± 7%, respectively) was higher than for polar body extruded embryos after 90 or 120 min (23 ± 5% and 14 ± 4% or 8 ± 1% and 4 ± 1%, respectively, *p* < 0.01), which were presumably >80% haploid.

Similarly, SCT embryos were activated with ionomycin and scored for extrusion of a pseudo-polar body. Unlike parthenogenotes, pseudo-polar bodies commonly contained all the DNA, fragmented into several chromatin masses (7/9 = 78% of PPB extrusions; Supplementary Figure S3F). Extrusion was assessed every 30 min and plateaued after around 90 min (*n* = 4, N = 104 embryos) but at levels below 60% (Supplementary Figure S3G). Embryos that had extruded a pseudo-polar body after 90 or 120 min were cultured in DMAP for 4 h, and development was assessed on D7 (Supplementary Figure S3H). Total and high-grade blastocyst development from standard controls (14 ± 4% and 8 ± 3%, respectively) was higher than for polar body extruded embryos after 90 or 120 min (3 ± 1% and 3 ± 1% or nil, respectively, *p* < 0.01). Reconstructs that did not extrude a pseudo-polar body after 120 min (*n* = 1, *N* = 19) were also cultured to D7, but no blastocyst development was observed. As delayed DMAP activation compromised *in vitro* development, we used standard chemical activation for subsequent cloning experiments.

Cloned *in vitro* development from embryonic mitotic donors was evaluated after standard chemical activation (Table 1). To improve *in vitro* development, we compared standard single cytoplast (“ECSC”) ECT with embryonic cell double cytoplast (“ECDC”) transfer, whereby another cytoplast was fused to the first ECT reconstruct. This had previously benefitted *in vitro* development across a range of somatic cell clones (Delaney et al., 2007; Green et al., 2015). ECDC doubled total blastocyst development and particularly increased the yield of high-quality blastocysts from mitotic donors. No significant difference in cleavage was observed, but total (18 ± 5% vs. 9 ± 4%; *p* = 0.03) and high-grade (9 ± 3 vs. 0%; *p* < 0.004) blastocyst development was higher with double compared to single cytoplast ECT, respectively.

**TABLE 1:** *In vitro* development of ECSC vs. ECDC embryos from mitotic ICM donor cells.

Cloning method	*n*	*N*	No. of ≥1-cells (% ± SEM)	No. of B1–3 (% ± SEM) ^+^	No. of B1–2 (% ± SEM) ^+^
ECSC	6	99	66 (67 ± 8%)^a^	9 (9 ± 4%)^a^	0^a^
ECDC	6	320	184 (58 ± 10%)^b^	59 (18 ± 5%)^b^	30 (9 ± 3%)^b^
*P*-value			ab = 0.134	ab = 0.034	ab = 0.004

Embryos were cloned from mitotic ICM donor cells, using either standard embryonic single cytoplast (ECSC) or sequential embryonic cell double cytoplast (ECDC) transfer; *n* = number of independent ECT experiments; ^†^percentage of embryos placed into IVC (N) that developed into D7 blastocysts (B) grade 1–3 (B^1-3^) or into B grade 1–2 (B^1-2^); ^a,b^ groups with these superscripts within a column differ by the indicated *p*-value, as determined by the two-tailed Fisher exact test in 2 × 2 tables.

### Mitotic donors produce karyotypically abnormal blastocysts

**FIGURE 4:**
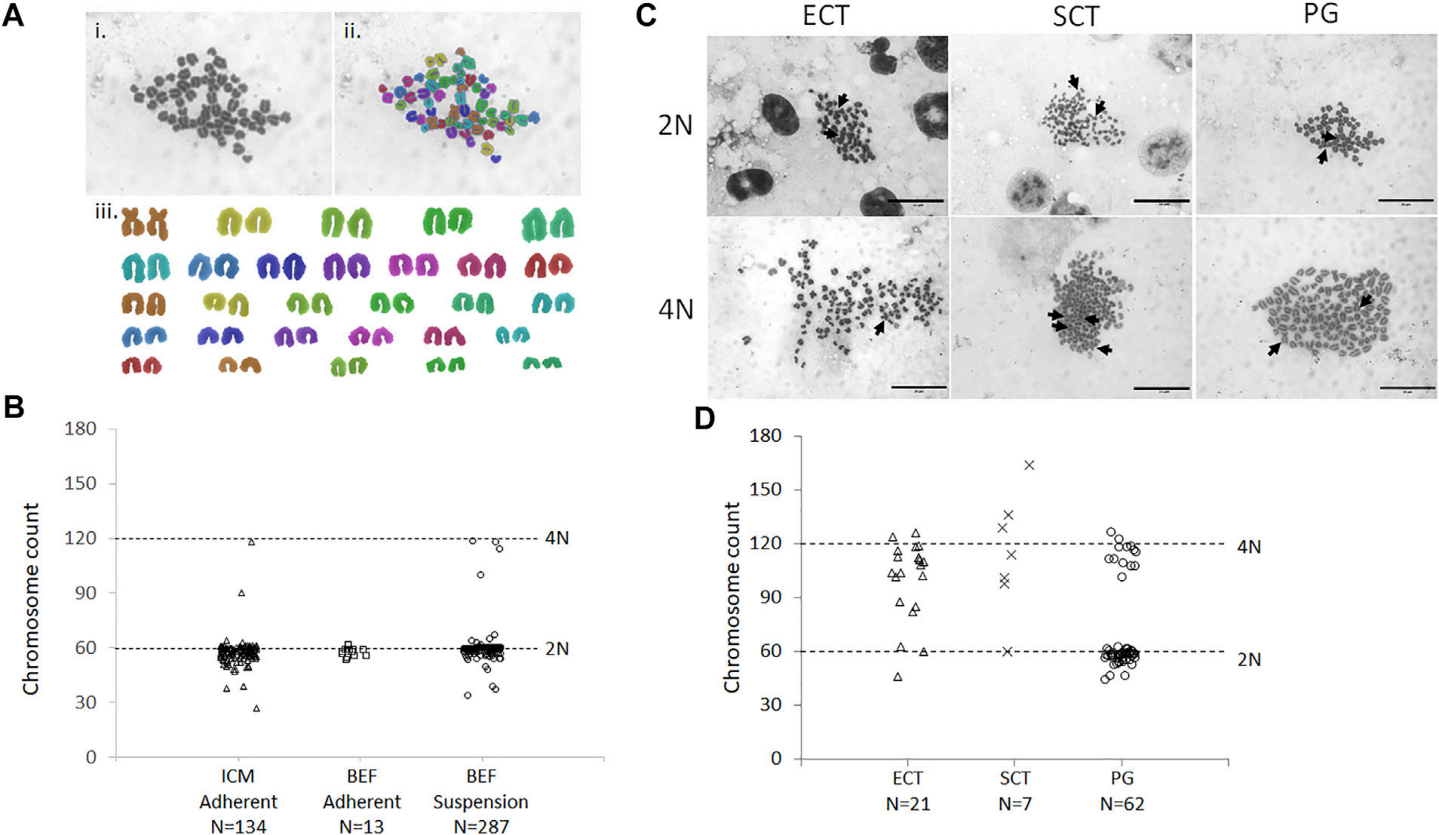
Karyotype of donor cells and ECT embryos. **(A)** Example karyotype of parthenogenetic embryo, containing 58 chromosomes, analyzed by SmartType. Image was taken at 1000× magnification. i) Individual chromosomes outlined using SmartType. ii) Chromosomes falsely colored for identification in the karyotype. iii) Chromosome karyotype separated from the background and arranged according to size. Metacentric sex chromosomes (brown) are paired at the top right. **(B)** Distribution of chromosome spreads from ICM and bovine embryonic fibroblasts (BEF), obtained by either *in situ* adherent or standard suspension karyotyping. **(C)** Cloned blastocysts were generated from ICM (ECT) or BEF (SCT) donors, and chemically activated parthenogenotes (PG) served as controls. 2N (diploid) and 4N (tetraploid) indicate approximately 60 and 120 chromosome counts, respectively. Arrows indicate metacentric sex chromosomes. Scale bar = 20 μm. **(D)** Distribution of chromosome spreads from cloned and PG embryos. Horizontal dashed lines indicate approximately diploid (60) and tetraploid (120) chromosome counts. *N* = number of metaphase spreads counted.

Nocodazole-arrested mitotic bovine donor cells have a normal diploid number of chromosomes (2N) but double the amount of nuclear DNA content (4C). To simplify analysis from a limited number of mitotic donor cells, we first developed a method to accurately karyotype adherent embryonic donor cells *in situ*. This method was based on our standard procedure to *in situ* karyotype cells within bovine blastocysts. Chromosome spreads were produced for adherent *in situ* vs. standard suspension karyotyping of somatic cells and analyzed using SmartType software (Figure 4A). Adherent embryonic fibroblasts showed a similar distribution as conventional suspension cells, with most chromosome counts clustered around the diploid number of 60 chromosomes (Figure 4B). Similarly, 85% of adherent ICM-derived cells had 60 ± 5% chromosomes (*N* = 28 colonies, *n* = 6 IVP runs). Since CHIR99021 has been associated with inducing chromosomal segregation errors (Tighe et al., 2007), we tested its effect on karyotypic abnormalities. Culturing cells in a CHIR99021-containing growth medium did not affect the proportion of diploid somatic (Supplementary Figure S4A) or ICM-derived donor cells, even after increasing culture from 6 to 14 days (Supplementary Figure S4B).

Next, karyotypes of cloned blastocysts from ICM donors and chemically activated parthenogenetic controls were analyzed in the same way (Figure 4C). A bimodal distribution was observed in parthenogenetic karyotypes, with chromosome spreads grouping around 2N/60 and 4N/120 chromosomes ± 10% (68 and 16% of spreads counted, respectively, Figure 4D). Chromosome spreads from cloned blastocysts had more varied chromosome numbers, with less clearly defined groups. Both SCT- and ECT-derived blastocysts presented with a small fraction of spreads around 2N/60 chromosomes (14 and 10%, respectively), but the majority contained 60–120 (29 and 33%, respectively) or 4N/120 and more chromosomes (52 and 58%, respectively). Thus, over 90% of ECT-derived blastocysts were tetraploid or aneuploid with extra chromosomes, compared to less than 2% (2/134 = 1.5%) in the original ICM donor cells.

### Cloned blastocysts from mitotic donors develop poorly *in vivo*

Last, we transferred cloned embryos from mitotic ICM donors and quiescent fibroblasts, as well as IVF controls, into surrogate dams. ECT embryos (*N* = 75), representing 28 different donor ICMs, were generated in seven different cloning runs, using either standard ECSC or sequential ECDC (Table 2). There was no difference between the two methods in initial pregnancy establishment at D35 (1/22 = 5% vs. 4/53 = 8% for ECSC vs. ECDC, respectively). A subset of ECT embryos (9/75 = 12%) was vitrified, including both ECSC vs. ECDC groups (7/22 = 32% vs. 2/53 = 4%, respectively), whereas all SCT and IVF controls were transferred freshly. However, vitrification does not compromise initial post-blastocyst survival in cattle (Fisher et al., 2012).

**TABLE 2:** *In vivo* development of ECT vs. SCT vs. IVF embryos.

Group	*n*	*N*	D35 (% ± SEM)	D56 (% ± SEM)	D83 (% ± SEM)
ECT	7	75	5 (7 ± 6%)^a^	1 (1 ± 1%)^a^	1 (1 ± 1%)^a^
SCT	2	11	3 (27 ± 6%)^b^	3 (27 ± 6%)^b^	2 (18 ± 5%)^b^
IVF	2	20	9 (45 ± 2%)^c^	9 (45 ± 2%)^c^	9 (45 ± 2%)^c^
*P*-value			ab = 0.124	ab = 0.012	ab = 0.084
			ac = 0.0003	ac = 0.000003	ac = 0.000003
			bc = 0.57	bc = 0.57	bc = 0.27

Embryos were cloned from nocodazole-arrested, mitotic ICM-derived primary cultures (ECT), serum-starved, quiescent adult ear skin fibroblasts (SCT) or *in vitro* fertilized controls (IVF). *n* = number of independent experiments; *N* = number of blastocysts transferred into surrogate dams; ^a,b,c^ = groups with these superscripts within a column differ by the indicated *p*-value, as determined by the Fisher exact test in 2 × 2 tables.

A subset of ECT pregnancies was tested for blood progesterone to determine a biochemical pregnancy at D21. By that stage, most recipients had returned to baseline progesterone levels, indicating that the majority of ECT blastocysts (31/39 = 80%) had already failed before uterine attachment. At D35, implantation was reduced in the ECT group when compared to both SCT and IVF controls (Table 2). This difference remained from D56 until D83 when all pregnancies were aborted due to animal welfare considerations. Overall, only one ECT pregnancy (1/75 = 1.3%) held until the end of the first trimester, compared to two (2/11 = 18%) from the SCT control group. We conclude that *in vivo* survival from mitotic embryonic donors was poor, probably due to ploidy errors arising during *in vitro* development of cloned embryos (Supplementary Figure S5).

## Discussion

Herein, we have shown that IL-6 supplementation benefits the establishment of primary bovine ICM cultures under feeder-free, chemically defined conditions. Mitotic shake-off allowed efficient isolation of single embryonic donor cells for ECT and the production of high-quality cloned blastocysts under optimized ECT conditions. However, most mitotic ECT-derived blastocysts were karyotypically abnormal, resulting in poor post-implantation development and reduced cloning efficiency in cattle.

### Role of interleukin signals in bovine ICM cells

Reports on the functionality of the IL-6/LIF-STAT3 pathway in bovine embryos are controversial. Human or mouse LIF either increased (Sirisathien et al., 2003; Wooldridge and Ealy, 2021a), decreased (Vejlsted et al., 2005) or did not affect blastocyst cell numbers (Rodriguez et al., 2007), while IL-6 more consistently increased ICM size (Wooldridge and Ealy, 2019; Wooldridge et al., 2019; Wooldridge and Ealy, 2021b). Bovine data may have been influenced by using heterologous cytokines, and we, therefore, first validated that commercially available, recombinant reagents were biologically active. Following cytokine-starvation of bovine cell lines and short-term stimulation with interleukin candidates (LIF, OSM, and IL-6), all three stimulated STAT3^Y705^ phosphorylation at the same concentration, indicating similar biological activity. Likewise, they increased the size of primary ICM colonies, consistent with their trophic effect on ICM cell numbers in embryos (Sirisathien et al., 2003; Wooldridge and Ealy, 2019; Wooldridge et al., 2019; Wooldridge and Ealy, 2021a; Wooldridge and Ealy, 2021b). These activities were not additive, indicating that they were likely acting through the same signaling pathway. Members of the IL-6 cytokine family (LIF, OSM, IL-11, IL-27, IL-31, ciliary neurotrophic factor [CNTF], cardiotrophin [CT], and cardiotrophin-like cytokine [CLC]) utilize a common signal-transducing receptor subunit (IL6ST or GP130) and ligand-specific receptor subunits to activate Janus kinase (JAK)-induced intracellular STAT3 phosphorylation (Rose-John and Family Cytokines, 2018). Several members (LIF, OSM, CNTF, and soluble IL6R) are functionally redundant in supporting naïve mouse ES cell culture (Nichols et al., 1994; Yoshida et al., 1994), and critical components of this pathway are operational within the bovine ICM (Meng et al., 2015; Wooldridge and Ealy, 2019; Wooldridge et al., 2019).

To gain a more comprehensive picture of interleukin signaling in bovine embryonic cultures, we quantified 76 interleukin signaling-related components and found that all candidates were detectable in bovine embryo cultures. About half of these sequences were low-to-medium abundant in DMSO and 2i+/IL-6, respectively, including several IL-6 family members (CNTF, OSM, IL6, IL-11/-11Ra, IL-27, IL-31/-31Ra, CT-1, and IL11/IL11R). The remainder was high to very highly expressed, including several receptors of the IL-6 family (IL-6R, IL-6ST, and IL-27Ra). These findings expand previous studies that have found a small subset of interleukin signaling components in bovine blastocysts, using either PCR (*IL6*, *IL6R*, and *IL6ST*) (Wooldridge and Ealy, 2019), (*LIFR* and *IL6ST*) (Eckert and Ni emann, 1998) or digital expression profiling (*IL6R* and *IL6ST*) (McLean et al., 2014). The receptors are usually expressed in the apical domain of epithelial cells, for example, on trophectoderm cells (Blomberg et al., 2008; Hall et al., 2009). In embryonic cell cultures, the basal surface is in contact with the laminin substrate, while the apical domain faces upward and is readily accessible for binding exogenous IL-6 in the culture medium.

### Metaphase arrest facilitates single-cell isolation

Apicobasal polarity not only defines cellular signaling properties but also constrains the ability to isolate intact single donor cells for ECT cloning. Like primed mouse PSCs, livestock ICM and epiblast cells develop hallmarks of epithelization, such as apicobasal polarity. They secrete basal membrane components and are connected by robust tight junctions and desmosomes with associated microfilaments (Talbot and Garrett, 2001). They critically depend on these cell-to-cell interactions and do not survive dissociation into single cells during passaging, using either enzymatic (e.g., trypsin, collagenase, and pronase) or chemical (e.g., EDTA) means (Keefer et al., 2007). Historically, primary outgrowth cultures of pig epiblasts, for example, will lyse after a 5-min exposure to Ca^2+^/Mg^2+^-free PBS. This makes single-cell isolation and clonal expansion of primary cultures difficult. By contrast, primary outgrowth of mouse ICMs or epiblasts are routinely dissociated with trypsin-EDTA, and their re-plating efficiencies are usually above 20% (Nagy and Vintersten, 2006). This resilience also enables the routine use of mouse ESCs for clonal expansion and chimera production through morula aggregation or blastocyst injection, both techniques that are still difficult with livestock ePSCs. It has even been suggested that the routine dissociation into single cells may break down cell-to-cell signaling that would otherwise promote ESC differentiation, thereby aiding in maintaining pluripotency during passaging (Talbot and Blomberg Le, 2008).

We applied the mitotic shake-off technique (Tobey et al., 1967) to obtain sufficient numbers of intact single ECT donors. Cells naturally increase their osmotic pressure and round off during mitosis (Stewart et al., 2011), minimizing their contact surface with the substrate and facilitating mechanical isolation. Subsequent positive selection of mitotic donors was through their maximal size compared to other cell cycle stages, which was easily identified by each operator under the stereomicroscope. Given their larger size and reduced scope for expansion, mitotic cells were more susceptible to lysis in the hypo-osmolar medium. Iso-osmolar buffer, combined with pronase treatment, led to acceptable ECT fusion rates above 75%.

### Nocodazole effects on *in vitro* development of cloned embryos

Preceding shake-off, chemical cell-cycle synchronization required the use of nocodazole to increase the yield of mitotic donors. This drug rapidly crosses membranes and binds tubulin, arresting cells in prometaphase by interfering with microtubule polymerization and the formation of metaphase spindles. In cultured cells, micromolar concentrations (>1 µM) caused complete depolymerization of microtubules, whereas substoichiometric nanomolar concentrations, relative to tubulin, only mildly altered microtubule and centrosome structure (Jordan et al., 1992). We chose 0.5 µM nocodazole to maximize the yield of mitotic donors, accepting that this may have subtly compromised centrosome structure and function following nocodazole removal (Jordan et al., 1992). Our choice was also based on previous blastomere cloning experiments that had identified 0.33 µM as the minimal concentration to achieve 100% cleavage arrest in mouse embryos (Samake and Smith, 1996). Most other groups have used 5–20-fold higher concentrations for >12 h and still observed high reversibility of the drug, resulting in the successful blastocyst and *in vivo* development in mouse (Kato and Tsunoda, 1992; Kwon and Kono, 1996), sheep (Liu et al., 1997), and cattle (Tanaka et al., 1995). Cells and donor-cytoplast couplets were kept in nocodazole until electrical fusion when they were washed out of the drug by >10,000-fold dilution in fusion buffer (>50 ml). From entering the fusion chamber until chemical activation and *in vitro* culture, the ECT reconstructs had about 1–5 h recovery time, respectively, which is comparable to previous studies that generated live offspring at micromolar nocodazole concentrations (Kato and Tsunoda, 1992; Tanaka et al., 1995; Kwon and Kono, 1996; Liu et al., 1997). Nevertheless, it is possible that prolonged nanomolar nocodazole exposure and carry-over from donor cells compromised further ECT embryo development. This is particularly relevant during cattle ECT, where the oocyte’s only endogenous microtubule-organizing center is removed during enucleation (Tani et al., 2007) and replaced by the nocodazole-treated centrosomes of the donor cell.

Cells in mitosis were assessed by Hoechst and P-H3 stain to identify condensed chromatin. At 500–1,670 nm nocodazole, about 37–49% of the cells in each colony were in mitosis. This number is probably an underestimate because mitotic cells are more easily lost from the colony during staining, owing to their relatively loose attachment to the substrate. Following chemical, enzymatic, and mechanical dissociation, the largest single cells within the population were selected for ECT to further enrich for mitotic cells. Following mitotic ECT, >90% of cloned blastocysts were tetraploid or aneuploid, which is compatible with the preferential selection of mitotic donors. Nevertheless, we cannot exclude that some non-mitotic donors were selected for SCT. Those donors would have been in G_0_/G_1_– or S-phase. G_0_/G_1_ donors can produce blastocysts and offspring upon transfer into MII cytoplasts, whereas S-phase donors cannot (Campbell et al., 1996b; Tani et al., 2001). The presence of S-phase donors could explain the relatively low *in vitro* development but would not affect *in vivo* development because S-phase donors are incompatible with producing blastocysts.

### Epigenetic reprogramming of mitotic donor cells to promote development

Under conditions that maintain normal ploidy, epigenetic effects of the donor cell cycle could influence nuclear reprogramming. Cell cycle-dependent epigenetic changes in chromatin status and gene expression include nuclear envelope assembly and disassembly, modifications of chromatin and chromatin-binding proteins, transcription of critical cell cycle regulators, accessibility of origins for DNA replication, and DNA repair. Using the genetically permissive G_2_/M phases of the cell cycle may promote epigenetic reprogramming by releasing chromatin-associated factors during chromosome condensation. After activation, ooplasmic factors may then more easily gain access to the decondensing chromatin during pseudo-pronuclear formation. There has been the suggestion that this may result in naturally higher reprogrammability of M-phase nuclei. For example, transcription factors are removed during M-phase chromatin condensation (Martinez-Balbas et al., 1995), somatic histone H1 is removed more quickly from metaphase donor nuclei following SCT (Bordignon et al., 2001), and re-expression of pluripotency genes occurs up to 100 times faster than for interphase nuclei (Halley-Stott et al., 2014). This “mitotic advantage” is thought to be related to the chromatin state itself, specifically the loss of histone H2A ubiquitination, rather than to accelerated access of reprogramming factors to the DNA, DNA modifications, or other post-translational histone modifications (acetylation, phosphorylation, and methylation). However, these systems relied on the interspecies transfer of permeabilized mouse myoblasts into *Xenopus* cytoplasts, using rapid expression of pluripotency genes as the main readout for reprogramming. Reprogramming efficiency, the product of recipient reprogramming ability and donor reprogrammability, can be quantified at different levels. This includes 1) changes in DNA and DNA-binding proteins, 2) termination of somatic and initiation of embryonic gene expression, and 3) progression to certain developmental milestones, such as blastocyst formation, implantation, and survival into adulthood. Among those, reprogramming into viable offspring is the most definitive measure of extensive donor cell reprogramming, referred to as cloning efficiency (Oback and Wells, 2007). Using this criterion, the significance of the postulated mitotic advantage for epigenetic reprogramming remains obscure. Whilst mitotic somatic cells can reprogram efficiently under certain activation conditions (see below), there is no indication that their cloning efficiency is higher than that of quiescent G_0_ cells, which also show features of elevated epigenetic reprogrammability (Kallingappa et al., 2016). We have not analyzed aberrant DNA and histone methylation patterns or dysregulated gene expression but instead focused on cloned embryo development as a functional bioassay for epigenetic reprogramming.

### Increasing reconstructed embryo volume improves *in vitro* development

To improve *in vitro* development, we compared standard single cytoplast with double cytoplast ECT transfer and noted a doubling of blastocyst development, especially into high embryos. This method had been originally developed to reconstitute the cytoplasmic volume after oocyte bisection and blastomere cloning, where it also increased embryo yield and quality (Peura et al., 1998). We extended this work to improve *in vitro* development and cloned embryo quality from eight somatic cell lines (Delaney et al., 2007). A similar effect was observed in interspecies SCT between domestic and Argali sheep, where double cytoplast SCT increased the proportion of high-quality embryos (Green et al., 2015). Importantly, this did not improve post-blastocyst development and pregnancy rates on D35 (Delaney et al., 2007). Likewise, we did not observe an improvement in initial pregnancy establishment at D35 and beyond between single and double cytoplast ECT in the current study. Thus, our results confirm a robust beneficial *in vitro* effect that was also evident in embryonic donors and hence conserved across the donor cell spectrum, ranging from blastomeres *via* embryonic cells to various somatic donors. The underlying molecular mechanism for this improvement will be investigated in future studies. Principally, this improvement could be due to doubling the amount of beneficial reprogramming activators or halving the concentration of reprogramming inhibitors.

### Genetic errors in embryos cloned from mitotic donors

Cell-cycle coordination between the nuclear donor and enucleated recipient cell maintains normal ploidy in the reconstructed cloned embryo (Campbell et al., 1996b). This depends on the activity of mitotic cyclin-dependent kinase (M-Cdk) in the cytoplast. M-Cdk activity is high in MII cytoplasts but declines after activation. When a mitotic donor nucleus is introduced into an MII cytoplast, both cells are fully compatible. The donor DNA is duplicated (4C), and sister chromatids cohere (2N) for equal segregation; the condensed chromatin is transcriptionally inactive; the nuclear envelope is dissociated; the centriole is replicated, and the mitotic spindle is formed. Following ECT and activation, the sister chromatids attach to opposite poles of the spindle and segregate regularly. In mouse cloning, this initiates expulsion of a pseudo-polar body and produces a diploid single pseudo-pronucleus. Mouse metaphase donors were first successfully used with 4-cell blastomeres (Kwon and Kono, 1996), followed by ES cells (Wakayama et al., 1999; Amano et al., 2001; Zhou et al., 2001) and fetal fibroblasts (Ono et al., 2001). This success has been attributed to the presence of multiple microtubule-organizing centers in mouse oocytes, which enable the extrusion of a pseudo-polar body (Tani et al., 2007). Correct chromatin configuration on a spindle, pseudo-polar body extrusion and development were improved when activation was delayed, enabling metaphase alignment (Cheong et al., 1993; Fulka et al., 1993; Alberio et al., 2000; Amano et al., 2001). Nevertheless, abnormal chromosomal segregation was frequent in cloned mouse embryos (Fulka et al., 1993; Ouhibi et al., 1996; Tani et al., 2007; Mizutani et al., 2012) and accounted for a larger effect on embryo viability than epigenetic reprogramming errors (Mizutani et al., 2012).

In ruminant oocytes, the cytoskeletal organization differs from mouse (Schatten, 1994). Following enucleation, the introduced donor cell provides the missing centrosome, which directs chromosomal segregation. Under these conditions, full-term development was first obtained after fusing bovine 16-cell blastomeres, synchronized with 10 μM nocodazole for 12 h, into aged MII cytoplasts (Tanaka et al., 1995). Live cloned sheep were also derived from fusing putative mitotic morula blastomeres, synchronized with 6.6 μM nocodazole for 14–17 h, into sheep MII cytoplasts (Liu et al., 1997). Attempts to clone bovine embryos from transferring mitotic blastomeres, synchronized with 1.2 μM nocodazole for 12–14 h, into MII cytoplasts and delayed chemical activation with ethanol and cycloheximide (CHX), initially failed (Alberio et al., 2000). By contrast, Heyman et al. injected M-phase nuclei and obtained live offspring but provided little experimental detail (Heyman et al., 2002b). Another study produced cloned blastocysts from injecting mitotic fetal fibroblasts, synchronized by methoxyestradiol, into MII cytoplasts and activating within 10 min by electrical pulse, ionophore, and CHX, but none of these blastocysts were reportedly transferred into surrogates (Ideta et al., 2005). Prior to both studies, Tani et al. produced both blastocysts and one live offspring from mitotic somatic donors (Tani et al., 2001). The authors synchronized cumulus cells with 10 μM nocodazole for 20–24 h and fused them with *in vitro* matured (22–24 h) MII cytoplasts. Single donors were morphologically confirmed to be in metaphase and electrically fused before exiting mitosis (<1 h). Reconstructs were electrically and CHX activated, scored for pseudo-polar body extrusion, *in vitro* cultured, and transferred. One out of five blastocysts from reconstructs with pseudo-polar body extrusion produced a live cloned calf. The two latter studies both used CHX activation without cytochalasin B and observed pseudo-polar body extrusion in about 30% of reconstructs, presumably restoring a diploid chromosome content. Development into blastocysts from this extrusion group was similar to the 70% of embryos that did not extrude a pseudo-polar body (Tani et al., 2001; Ideta et al., 2005). Both studies claimed that between 75% (Ideta et al., 2005) and 100% (Tani et al., 2001) of blastocysts from the non-extruded group were tetraploid but did not report detailed evidence from their respective chromosome analysis. Our karyotyping data strongly support the notion that reconstructs without a pseudo-polar body will develop into tetraploid blastocysts. Using chemical activation with DMAP, a broad-spectrum kinase inhibitor that prevents pseudo-polar body expulsion, produced over 90% of blastocysts with cells carrying extra chromosomes or tetraploid content. This compound is also mutagenic in the micromolar range (Katoh et al., 2004). We nevertheless chose DMAP because blastocyst development was almost two-fold higher than using CHX in our hands, both for parthenogenotes and clones (data not shown), maximizing the biological amplification of potentially valuable blastocysts, at least *in vitro*.

In parthenogenotes, delaying the addition of DMAP after ionomycin has resulted in high second polar body extrusion, formation of a single pronucleus, and production of a high proportion of haploid activated oocytes (Rho et al., 1998). Our attempts to combine pseudo-polar extrusion with DMAP activation were less successful. Following ionomycin activation, we withheld DMAP for 90 min to allow pseudo-polar body extrusion in about 50% of the reconstructs. This proportion was similar to embryos extruding polar body-like structures after delayed activation (3–7 h post-fusion) of reconstructs with mitotic donors (Alberio et al., 2000). Delayed DMAP resulted in a >2.5-fold decrease in blastocyst development when compared to embryos that were exposed to DMAP within 10 min. This decrease in cloned embryo development was likely due to two factors: 1) pseudo-polar bodies frequently contained all the donor DNA, often fragmented, leaving an empty cytoplast that would be unable to develop, and 2) 90 min was sufficient time for some M-Cdk complexes to reform in the absence of DMAP. This would prevent nuclear membrane reformation, interrupt resumption of the cell cycle, and compromise the development of all reconstructs irrespective of whether they had correctly expelled a pseudo-polar body. Oocytes resumed meiosis and formed multiple pronuclei or polar bodies after ionomycin activation for an hour without DMAP exposure, indicating that ionomycin treatment alone is not sufficient to ensure correct chromatin reorganization (Susko-Parrish et al., 1994). Our data are consistent with findings that ionomycin/DMAP increases the proportion of mixoploid or polyploid SCT blastocysts when compared to ionomycin alone or ionomycin/CHX (Bhak et al., 2006).

### Cloned blastocysts from mitotic donors develop poorly *in vivo*

Most cloned blastocyst chromosome spreads analyzed contained >100 chromosomes. Based on an average of 1.6 metaphase spreads per embryo (range 1–3), 90% of cloned blastocysts contained aneuploid or tetraploid cells. In order to determine whether the embryos were truly tetraploid or in fact mixoploid, more sensitive cytogenetic detection methods, such as karyotyping based on interphase FISH, would be needed (Viuff et al., 1999). To evaluate their potential for *in vivo* development, we transferred these embryos into surrogates. One pregnancy held until D84 when it was terminated, but unfortunately, the aborted fetus was not collected for karyotyping and ploidy determination. In previous work with diploid vs. tetraploid cloned blastocysts, generated by single or double SCT, respectively, we found a trend toward diploid embryos developing better to mid-gestation (Green et al., 2007). Despite double SCT blastocysts being tetraploid at the time of transfer, we observed that muscle and lung fibroblast cell lines derived from a double SCT fetus at D163 were diploid, indicating that ploidy correction might have occurred during development (Green et al., 2007). Alternatively, it is possible that tetraploid cells were preferentially allocated to the extraembryonic membranes, as in the case of mixoploid blastocysts in mice (James et al., 1995) and cattle (Viuff et al., 2002). By this mechanism, aneuploidies arising later in preimplantation development can be compensated through cells with the correct chromosomal constitution and produce viable cloned mice (Mizutani et al., 2012). However, bona fide tetraploid embryos exhibit a high degree of post-implantation abnormalities (Kaufman and Webb, 1990; Teyssier et al., 1997). Given these non-reprogrammable genetic errors, it is plausible that epigenetic reprogramming strategies, such as ECDC, did not achieve any developmental improvement. We conclude that ploidy errors arising from artificial activation with DMAP compromised *in vivo* survival of mitotic embryonic donors. This could be overcome by using alternative artificial activation methods and careful screening for pseudo-polar body extrusion, potentially allowing efficient multiplication of valuable embryonic genotypes by ECDC. Combined with embryo-mediated genome editing, this would allow us to selectively introduce naturally occurring beneficial variants into elite genetic backgrounds in a single generation.

## Data Availability

The original contributions presented in the study are included in the article/Supplementary Materials; further inquiries can be directed to the corresponding author.

## Supplementary material

Supplementary Figure S1 Recombinant cytokines are functionally active in bovine cells and support blastocyst plating and outgrowth expansion. **(A)** Bovine embryonic fibroblasts were serum starved overnight in 0.5% FBS and treated with LIF, OSM, IL-6 or water for 10 min. Fixed cells were stained with Hoechst 33342 (DNA) and anti-phosphorylated STAT3 (P-STAT3) antibodies. **(B)** Inner cell mass (ICM) plating efficiency was determined after one week in 2i+ medium, supplemented with LIF, OSM, IL-6 or water; n=number of biological replicates, P-values determined by 2-tailed Fisher exact test in 2X2 tables. **(C, D)** Colony area was determined after one week in 2i+ medium, supplemented with LIF, OSM, IL-6 or water **(C)**, as well as after one week in medium containing CHIR99021 and IL-6 with additional PD and LIF or OSM **(D)**; Error bars indicate mean ± SEM; a,b treatments differ by *p* < 0.05 from two-tailed *t*-test. *(E)*. On average, 93 ± 5% of ICMs were immunosurgically isolated from the blastocyst and 94 ± 2% of ICMs attached to the substrate, resulting in 87 ± 6% of viable primary colonies after one week (*N* = 105, *n* = 5). Error bars indicate mean ± SE.

Supplementary Figure S2 Optimisation of nocodazole synchronisation time **(A,B)** and concentration **(C,D)**. **(A)** Phase contrast and Hoechst stained (DNA) images of ICM colonies following treatment with DMSO for 21 h or 1.67 µM nocodazole (noco) for 4 or 21 h. Circles identify mitotic nuclei. **(B)** Quantification of mitotically arrested cells via chromatin condensation. Two colonies were assessed per treatment (*N* = 2), with an average of 114 nuclei counted per colony (44-193 nuclei per colony, total cells counted = 681). **(C)** Brightfield and Hoechst stained (DNA) images of ICM colonies arrested with 0.1-1.67 µM nocodazole for 24 h. **(D)** Quantification of mitotically arrested cells via chromatin condensation. A minimum of two colonies were counted per treatment (*N* = 2-4), with an average of 144 nuclei counted per colony (56-305 nuclei per colony, total cells counted = 1724). For all panels scale bar = 200 µm, error bars = SEM, and significance * = *p* < 0.05 and ** = *p* < 0.01 from two-tailed t-test compared with DMSO or 0.1 µM nocodazole.

Supplementary Figure S3 Optimisation of fusion and activation parameters.** (A**) Lysis in hypoosmolar vs isoosmolar fusion buffer. ** = treatments differ *P* < 0.01; *N=*494 couplets.** (B)** Effect of pronase in different fusion buffers. ** = treatments in the same buffer differ *P* < 0.01; *N=*611 couplets; error bars = SEM; n = number of replicates;** (C-H)** Polar body extrusion and *in vitro* development of parthenogenotes and cloned embryos after delayed DMAP exposure. **(C-E)** Parthenogenotes and **(F-H)** SCT embryos. **(C)** Telophase II stage oocyte 90 min after ionomycin activation. Arrows indicate second polar body (PB) with one half of Hoechst-stained DNA remaining inside the oocyte and one half being extruded. Remainder of chromatin was located within oocyte. Scale bar = 100 µm. **(D)** Average second polar body extrusion rate at 3, 15, 30, 45, 60, 75, 90, 105, 120, 150 and 180 minutes following ionomycin activation; n=8 experiments, N=557 oocytes analysed. **(E)** Development into D7 blastocysts of morphological grade 1-3 (B1-3) or 1-2 (B1-2). ** = P < 0.01 from two-tailed t-test compared with control activation (10 min). Error bars = SEM, *N*=517. **(F)** Telophase II stage SCT reconstructs 90 min after ionomycin activation. Arrows indicate pseudo-polar body (PPB) with all Hoechst-stained DNA being extruded. Scale bar = 100 µm. **(G)** Average pseudo-polar body extrusion rate at 3, 30, 60, 90, 120, 150 and 180 minutes following ionomycin activation; n=4 experiments, N=104 oocytes analysed. **(H)** Development into D7 blastocysts of morphological grade 1-3 (B1-3) or 1-2 (B1-2). N=134.

Supplementary Figure S4 Effect of CHIR99021 exposure on donor cell karyotype. Average proportion of diploid metaphase spreads (± 10%) from **(A)** bovine embryonic fibroblasts (BEF) cultured in DMEM/F12-based growth medium for six days with or without CHIR99021 and **(B)** from ICM-derived colonies cultured in 2i+ or N2B27 base medium for 6 vs. 14 days with or without CHIR99021; N = number of metaphase spreads counted.

Supplementary Figure S5 Predicted karyotype after mitotic ECT. Homologous chromosomes are shown in red and black. C = nuclear DNA content, N = nuclear chromosome number. Following artificial activation with 6-DMAP, the ECT reconstructs do not extrude a pseudo polar body, potentially resulting in tetraploid (4N) blastocysts (top path). In the absence of 6-DMAP, the ECT reconstruct commonly extrudes all chromosome (bottom path). Extrusion of a diploid (2N) pseudo-polar body was not observed (middle path).
