# Supplementary figures and images for: Double cytoplast embryonic cloning improves in vitro but not in vivo development from mitotic pluripotent cells in cattle
Source: Front Genet. 2022 Sep 28;13:933534. doi: 10.3389/fgene.2022.933534 (PMC9563626; doi:10.3389/fgene.2022.933534)

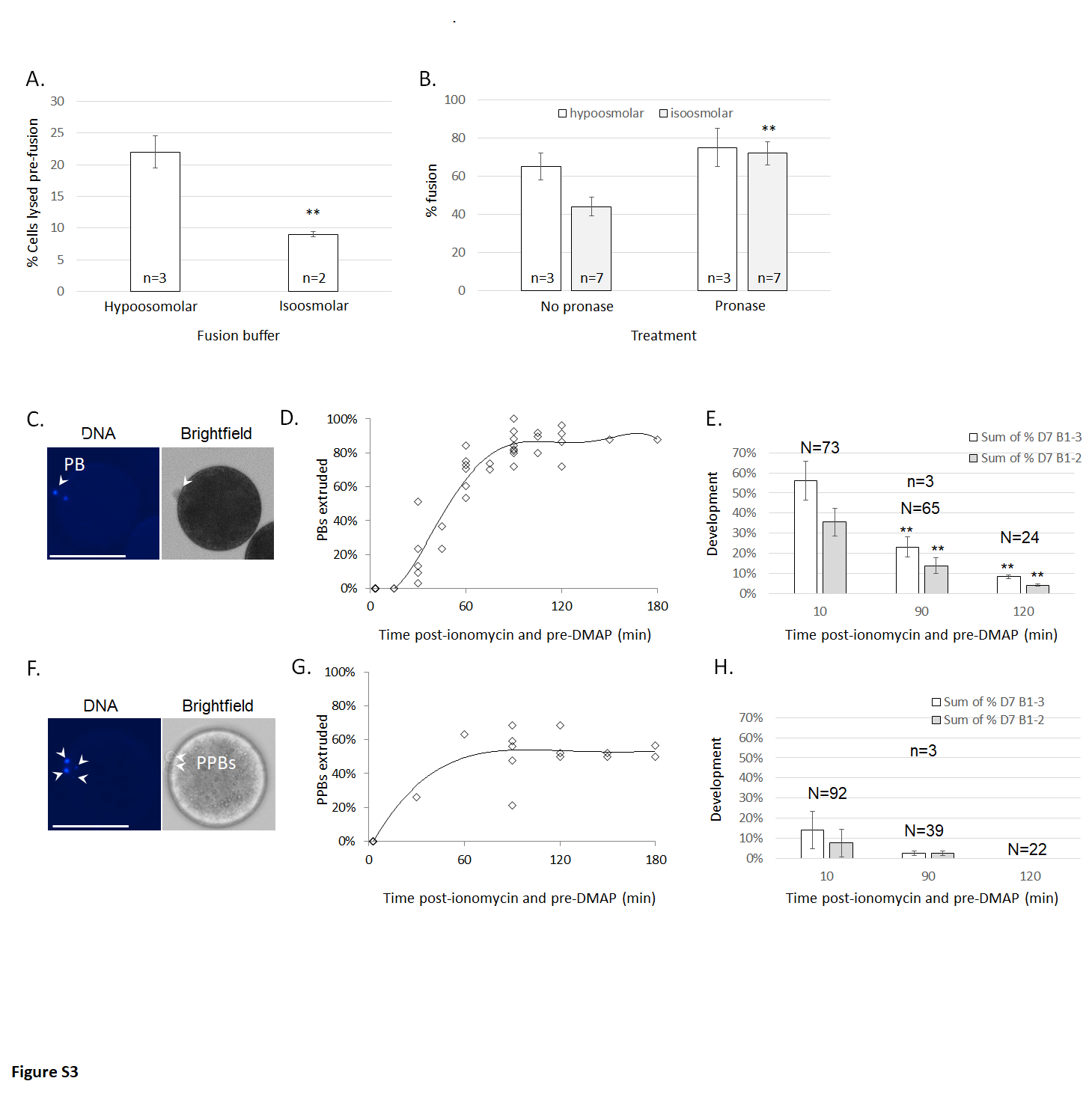

Supplement: Supplementary file 1 [file Image3.tif]

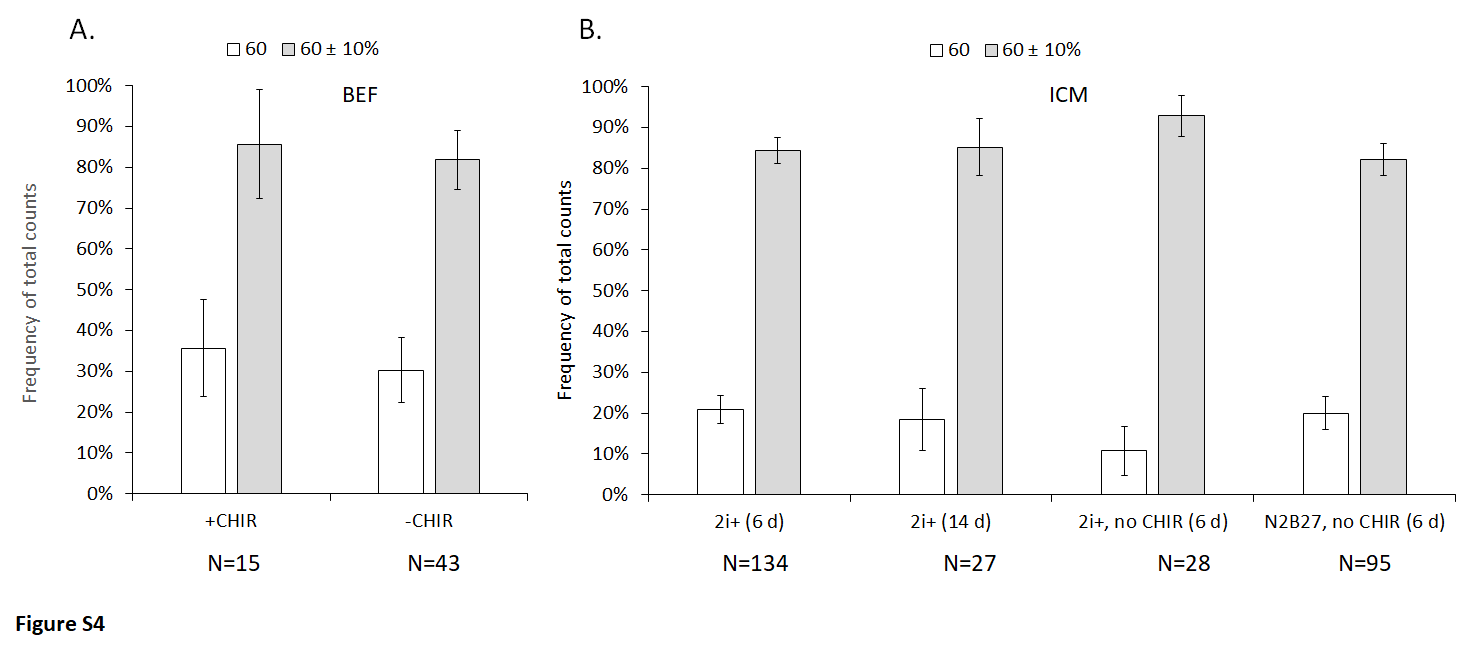

Supplement: Supplementary file 2 [file Image4.tif]

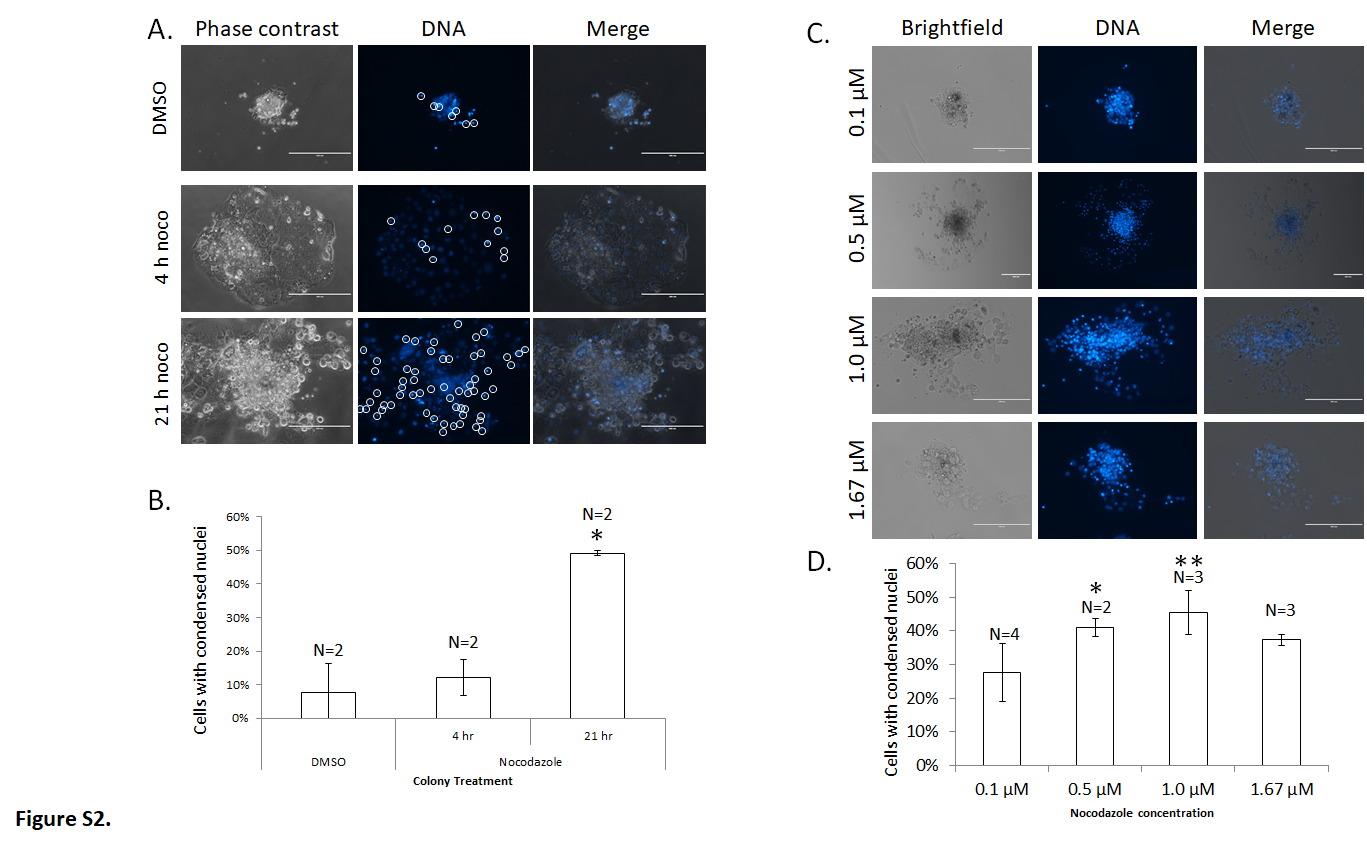

Supplement: Supplementary file 3 [file Image2.tif]

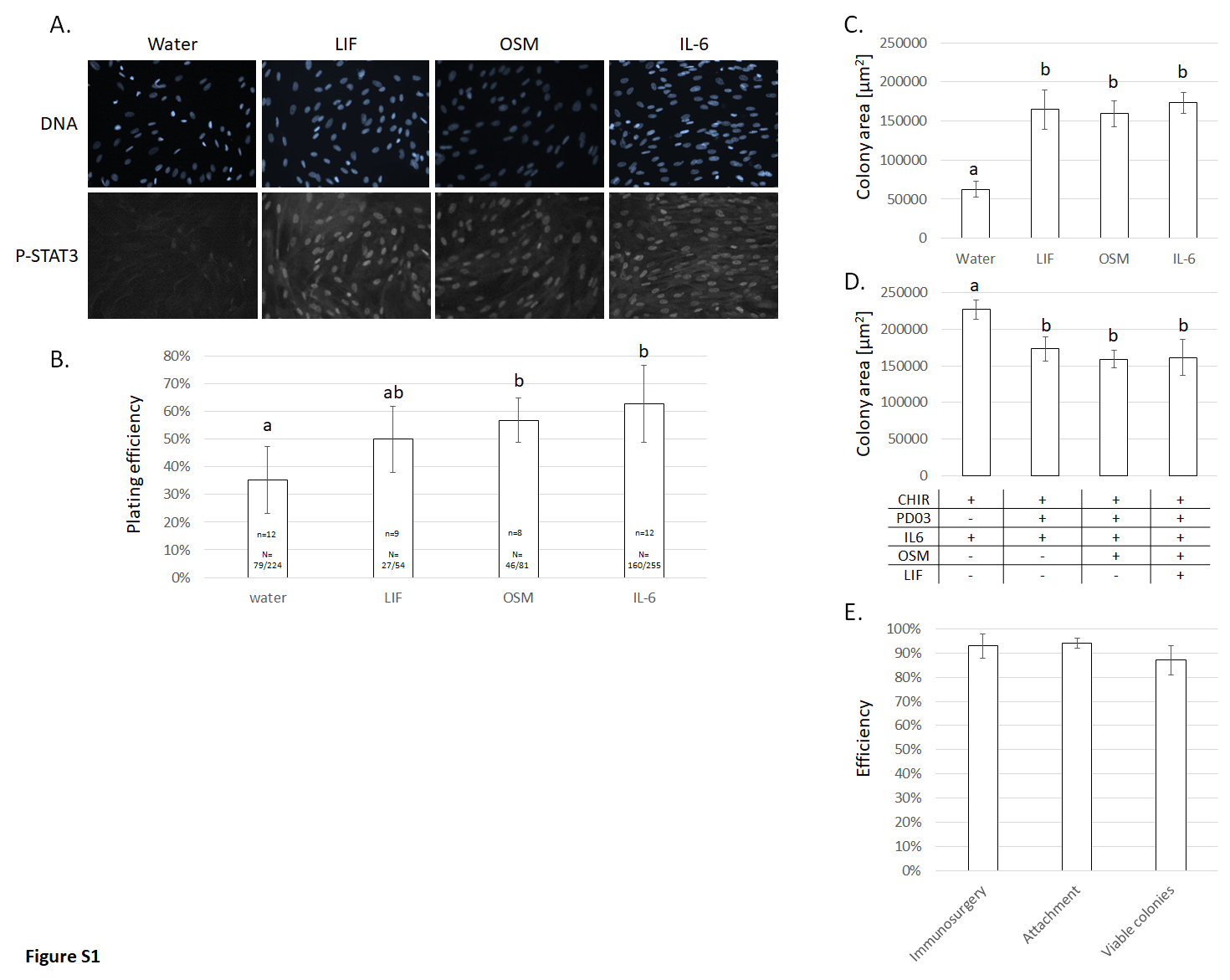

Supplement: Supplementary file 4 [file Image1.tif]

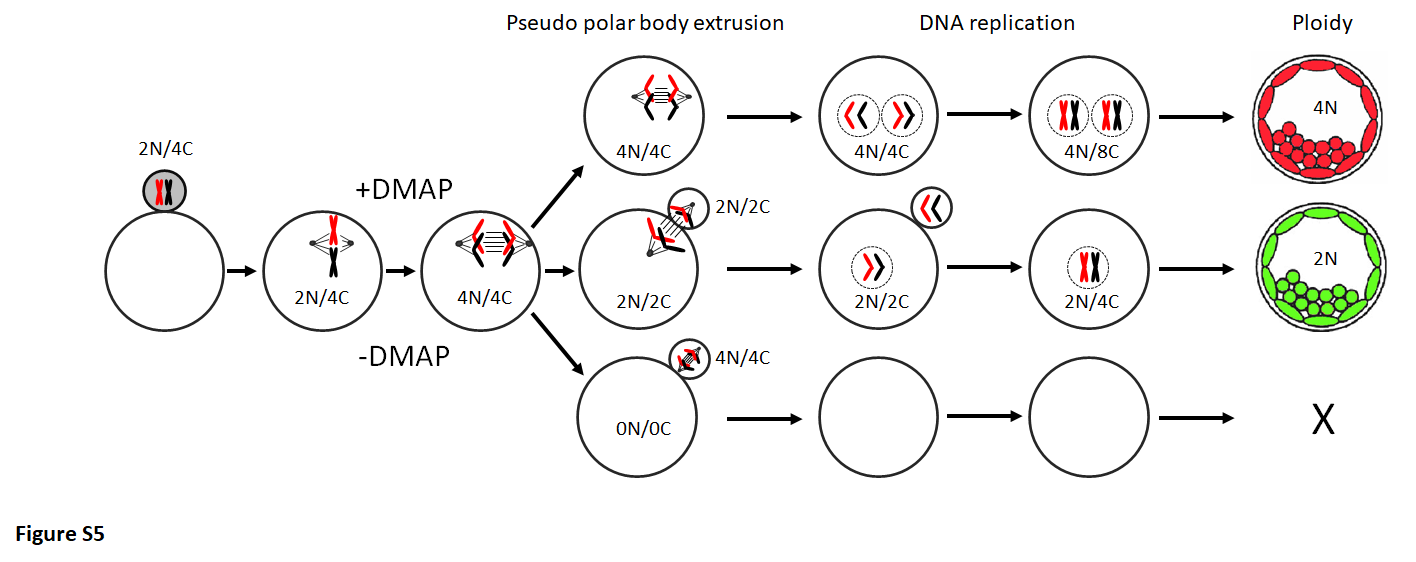

Supplement: Supplementary file 5 [file Image5.tif]
